# lncRNA CISAL Inhibits BRCA1 Transcription by Forming a Tertiary Structure at Its Promoter

**DOI:** 10.1016/j.isci.2020.100835

**Published:** 2020-01-11

**Authors:** Song Fan, Tian Tian, Xiaobin Lv, Xinyuan Lei, Zhaohui Yang, Mo Liu, Faya Liang, Shunrong Li, Xiaofeng Lin, Zhaoyu Lin, Shule Xie, Bowen Li, Weixiong Chen, Guokai Pan, Xinyu Lin, Zhanpeng Ou, Yin Zhang, Yu Peng, Liping Xiao, Lizao Zhang, Sheng Sun, Hanqing Zhang, Sigeng Lin, Qunxing Li, Binghui Zeng, Filippos Kontos, Yi Ruan, Soldano Ferrone, Dechen Lin, Bakhos A. Tannous, Jinsong Li

**Affiliations:** 1 Guangdong Provincial Key Laboratory of Malignant Tumor Epigenetics and Gene Regulation of Sun Yat-Sen Memorial Hospital, Guangzhou 510120, China; 2 Department of Oral and Maxillofacial Surgery, Sun Yat-Sen Memorial Hospital of Sun Yat-Sen University, Guangzhou 510120, China; 3 Department of Neurobiology, Key Laboratory of Human Functional Genomics of Jiangsu, Nanjing Medical University, Nanjing, Jiangsu 211166, China; 4 Nanchang Key Laboratory of Cancer Pathogenesis and Translational Research, Center Laboratory, the Third Affiliated Hospital, Nanchang University, Nanchang 330047, China; 5 State University of New York at Stony Brook, Stony Brook, NY 11794, USA; 6 Department of Stomatology, Longgang Distric Central Hospital, Affliated to Guangzhou University of Traditional Chinese Medicine, Shenzhen, Guangdong 518116, China; 7 Massachusetts General Hospital Cancer Center, Harvard Medical School, Boston, MA 02114, USA; 8 Department of Oral and Maxillofacial Surgery, Hainan General Hospital, Hainan 570300, China; 9 Guanghua School of Stomatology, Hospital of Stomatology, Guangdong Provincial Key Laboratory of Stomatology, Sun Yat-sen University, Guangzhou 510055, China; 10 Department of Surgery, Massachusetts General Hospital, Harvard Medical School, 55 Fruit Street, Boston, MA 02114, USA; 11 Department of Oral Medicine, Sun Yat-Sen Memorial Hospital of Sun Yat-Sen University, Guangzhou 510120, China; 12 Experimental Therapeutics and Molecular Imaging Lab, Department of Neurology, Massachusetts General Hospital and Harvard Medical School, Boston, MA 02129, USA

**Keywords:** Biological Sciences, Molecular Biology, Cell Biology, Cancer

## Abstract

Cisplatin-based neoadjuvant chemotherapy has been shown to improve survival in patients with squamous cell carcinoma (SCC), but clinical biomarkers to predict chemosensitivity remain elusive. Here, we show the long noncoding RNA (lncRNA) LINC01011, which we termed cisplatin-sensitivity-associated lncRNA (CISAL), controls mitochondrial fission and cisplatin sensitivity by inhibiting BRCA1 transcription in tongue SCC (TSCC) models. Mechanistically, we found CISAL directly binds the BRCA1 promoter and forms an RNA-DNA triplex structure, sequestering BRCA1 transcription factor-GABPA away from the downstream regulatory binding region. Importantly, the clinical relevance of these findings is suggested by the significant association of CISAL and BRCA1 expression levels in TSCC tumors with neoadjuvant chemosensitivity and overall survival. We propose a new model where lncRNAs are tethered at gene promoter by RNA-DNA triplex formation, spatially sequestering transcription factors away from DNA-binding sites. Our study uncovers the potential of CISAL-BRCA1 signaling as a potential target to predict or improve chemosensitivity.

## Highlights

• CISAL enhances mitochondrial fission and cisplatin sensitivity in TSCC cells through BRCA1
• CISAL directly binds the BRCA1 promoter and forms an RNA-DNA triplex structure
• CISAL sequesters GABPA away from regulatory binding at BRCA1 promoter
• High CISAL predicts favorable neoadjuvant chemosensitivity and prognosis of TSCC patients

## Graphical Abstract

**Figure:**
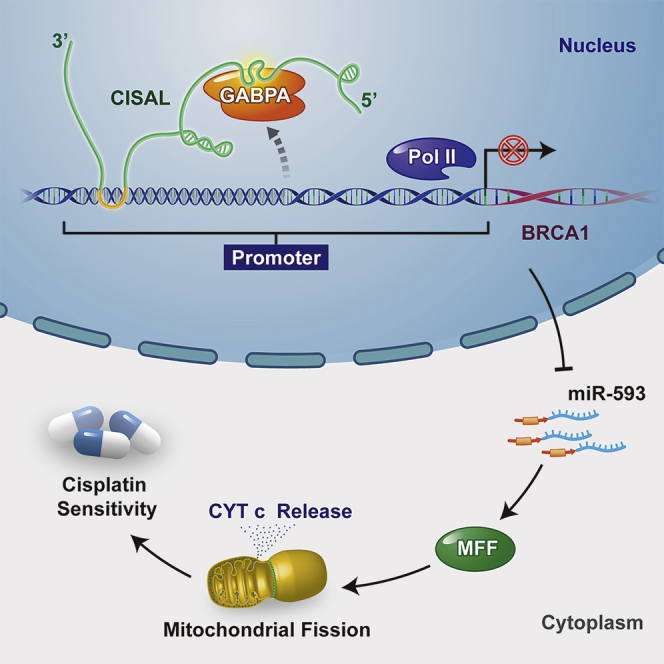

## Introduction

Around 70% of the human genome is transcribed to RNA, whereas only 2% consists of protein-coding genes (Ulitsky and Bartel, 2013). The many noncoding RNA (ncRNA) loci interdigitate between, within, and among protein-coding genes on both strands. In addition to miRNAs, recent studies suggest that long noncoding RNAs (lncRNAs), >200 nucleotides (nt), are expressed at lower levels than protein-coding transcripts and are more tissue specific (Cabili et al., 2011, Derrien et al., 2012). Further, lncRNAs can serve as scaffolds or guides to regulate protein-protein or protein-DNA interactions as decoys to bind proteins or microRNAs (miRNAs) and as enhancers to influence gene transcription when transcribed from enhancer regions (enhancer RNA) or their neighboring loci (ncRNA activator) (Hu et al., 2014). These lncRNAs have emerged as key regulators of important biological processes implicated in cell proliferation (Bian et al., 2018), differentiation (Russell et al., 2015), migration (Wang et al., 2017), immune response (Heward and Lindsay, 2014), and apoptosis in various cancer types, acting as tumor suppressors or oncogenes (Liu et al., 2018, Xing et al., 2018).

Mechanistically, recent studies revealed that a subset of lncRNAs regulate gene expression in *cis* and in *trans* by interacting with chromatin and recruiting chromatin modifiers (Bonasio and Shiekhattar, 2014, Carpenter et al., 2013). LncRNAs can function at their sites of synthesis to regulate local gene expression using either RNA-dependent (Lam et al., 2013) or RNA-independent mechanisms (Hah et al., 2013). A small but growing number of lncRNAs have been shown to positively regulate the expression of neighboring protein-coding genes on the same chromosome by altering local chromatin accessibility and/or structure (Lam et al., 2013, Melo et al., 2013). Furthermore, genomic binding profiles showed that a single lncRNA transcript can interact with multiple binding sites on different chromosomes away from its site of transcription (Rinn et al., 2007). Long-range intrachromosomal interactions between lncRNA-expressing loci and distant loci have also been documented (Hacisuleyman et al., 2014, Li et al., 2013). Although lncRNA can have dual functions, both acting locally to regulate the expression of its neighboring protein-coding gene and distally at regulatory elements genome-wide, the activity of lncRNAs depends largely on protein partners, such as transcription factors (TFs) or histones. Some studies suggested that lncRNAs could spatially correlate with TFs across the genome (Herriges et al., 2014), whereas others showed that lncRNAs appear to inhibit DNA binding of their associated TFs at several target sites (Sun et al., 2013). In any case, the direct interaction of lncRNA-DNA triplex and TFs remains unclear.

Recent studies revealed that an abnormal mitochondrial dynamic participates in the regulation of apoptosis (Suen et al., 2008) and is linked to a variety of diseases including cancer (Wang et al., 2011). Cisplatin has been heavily employed as a cornerstone treatment in the fight against a wide spectrum of solid neoplasms over the past 30 years. However, chemoresistance frequently develops and leads to therapeutic failure. The initial patient responsiveness to platinum-based therapies in oral squamous cell carcinoma (OSCC) is 80.6% (Zhong et al., 2013); however, more than 70% of patients eventually relapse due to tumor-acquired resistance (Gibson et al., 2005). Numerous studies tried to unravel the mechanism responsible for cisplatin resistance, but no substantive progress has been made to date to overcome this resistance. Here, we investigated the role of lncRNAs in regulating mitochondrial fission and cisplatin sensitivity in tongue squamous cell carcinoma (TSCC). We identified CISAL as one key lncRNA that participates in this process. Moreover, we show that CISAL can directly form an RNA–DNA triplex structure at the BRCA1 promoter and inhibit BRCA1 transcription activity by sequestering TF-GABPA away from its DNA-binding sites. Our data reveal a new role of lncRNAs in transcriptional regulation by expanding the known functions of the lncRNA-BRCA1 signaling axis in the mitochondrial network and chemosensitivity.

## Results

### Differential Expression of lncRNAs Induced by Cisplatin in TSCC Cells and Tumor Tissues

**Figure 1:**
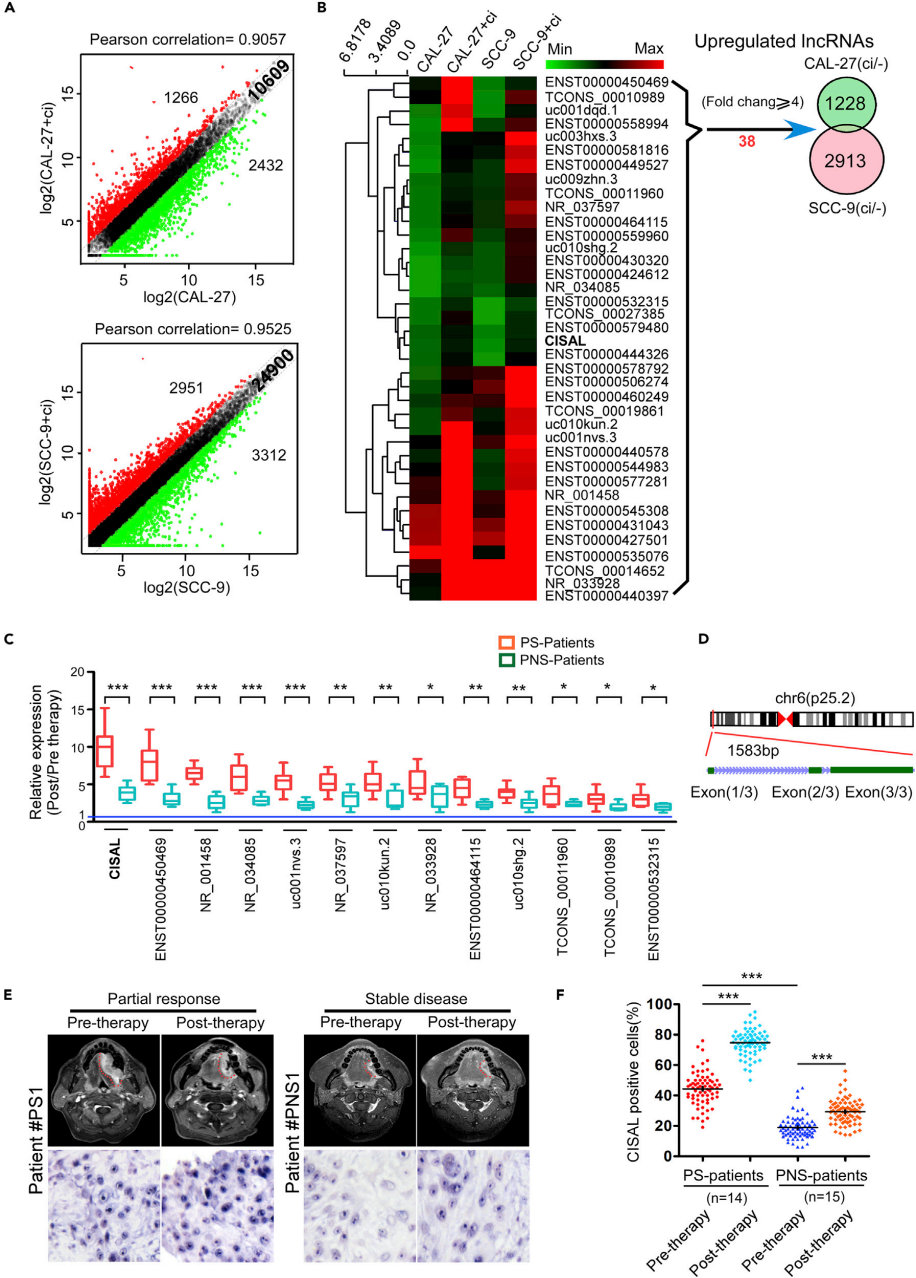
Differential Expression of lncRNAs Induced by Cisplatin in TSCC Cells and Chemosensitive or Chemoresistant Tumors (A) Scatterplot showing variation in lncRNA expression between treated and non-treated TSCC cells. The values on the X- and Yaxes are the average normalized signal values of the group (log2 scaled). The green and red dots represent fold change ≥2.0. (B) Heatmap showing 38 upregulated lncRNAs in both treated TSCC cell lines compared with the untreated cells. The relative lncRNA expression is depicted according to the color scale. Venn diagram of the intersection of the upregulated lncRNAs in both treated TSCC cell lines versus the untreated cells. Thirty-eight lncRNAs showed a fold change ≥4.0. (C) Thirteen lncRNAs were identified to be significantly upregulated in TSCC tumors with chemosensitive (PS) and chemoresistant tumors (PNS). (D) Schematic annotation of the CISAL genomic locus on chr6:2,988,648-2,991,173 in humans. Green rectangles represent exons. (E) Representative MRI scans of tumor response (upper panels) and CISAL expression (lower panels) in tissue specimens from patient with chemosensitive and chemoresistant TSCC tumors. (F) CISAL expression in each patient was analyzed by *in situ* hybridization; 5×200 tumor cells were randomly counted in each tumor. *p<0.05, **p<0.01, and ***p<0.001 versus control, (C) 2-tailed Student's t test; (F) 1-way ANOVA followed by Dunnett's tests for multiple comparisons.

Recent studies have demonstrated that lncRNAs play pivotal roles in regulating the biological properties of cancer (Peng et al., 2017). Previously, we showed that mitochondrial fission determines cisplatin sensitivity in tongue squamous cell carcinoma (TSCC) (Fan et al., 2015a, Fan et al., 2015b). We wonder whether lncRNAs participate in this chemosensitivity program in TSCC. We first profiled the expression of lncRNAs in two TSCC cell lines (CAL-27 and SCC-9) under cisplatin treatment in comparison to their matched untreated controls using microArray. A total of 1,266 upregulated lncRNAs and 2,432 downregulated lncRNAs with significant differential expression (fold change≥2) were found in CAL-27 cells, whereas SCC-9 cells showed 2,951 upregulated and 3,312 downregulated lncRNAs (Figure 1A). When we increased the cut-off for differentially expressed lncRNAs to ≥4, we found 38 upregulated lncRNAs (Figure 1B) and 143 downregulated lncRNAs (Figure S1A) under cisplatin treatment in both cell lines compared with their untreated parental controls. We then focused on these 38 upregulated lncRNAs and confirmed their expression levels in TSCC cells using qRT-PCR (Figure S1B). We also obtained matched pre- and post-cisplatin- treated TSCC tumor tissues from patients with chemosensitive and chemoresistant tumors (Table S1) and analyzed them for these lncRNA by qRT-PCR. Among the 38 lncRNAs, we found 19 of them to be highly upregulated in chemosensitive TSCC tumors before neoadjuvant chemotherapy as compared with chemoresistant tumors (Figure S1C) (fold change≥2). On the other hand, 13 lncRNAs were confirmed to be significantly upregulated in chemosensitive tumors, as compared with their matched pre-treated tumors as well as chemoresistant tumors (Figure 1C). Notably, one lncRNA (RefSeq accession number LINC01011) was mostly upregulated in chemosensitive tumors (Figures 1C and S1C). Thus, we focused on this uncharacterized lncRNA and named it CISAL (cisplatin sensitivity-associated lncRNA). We first confirmed that CISAL is located on chromosome 6 in humans and composed of three exons with a full length of 1583 nt by rapid amplification of cDNA ends (RACE) in the CAL-27 cell line (Figures 1D and S2A, and Table S2). The non-coding nature of CISAL was confirmed by coding-potential analysis (Figures S2B and S2 C). Expression of CISAL, further determined by locked nucleic acid (LNA)-based *in situ* hybridization (ISH), was markedly increased in patients with neoadjuvant chemosensitive tumors and was mainly localized to the nucleus (Figures 1E and 1F). The specificity of CISAL probes was confirmed as shown in Figures S3A–S3D.

### CISAL Regulates the Mitochondrial Fission and Cisplatin Sensitivity in TSCC

**Figure 2:**
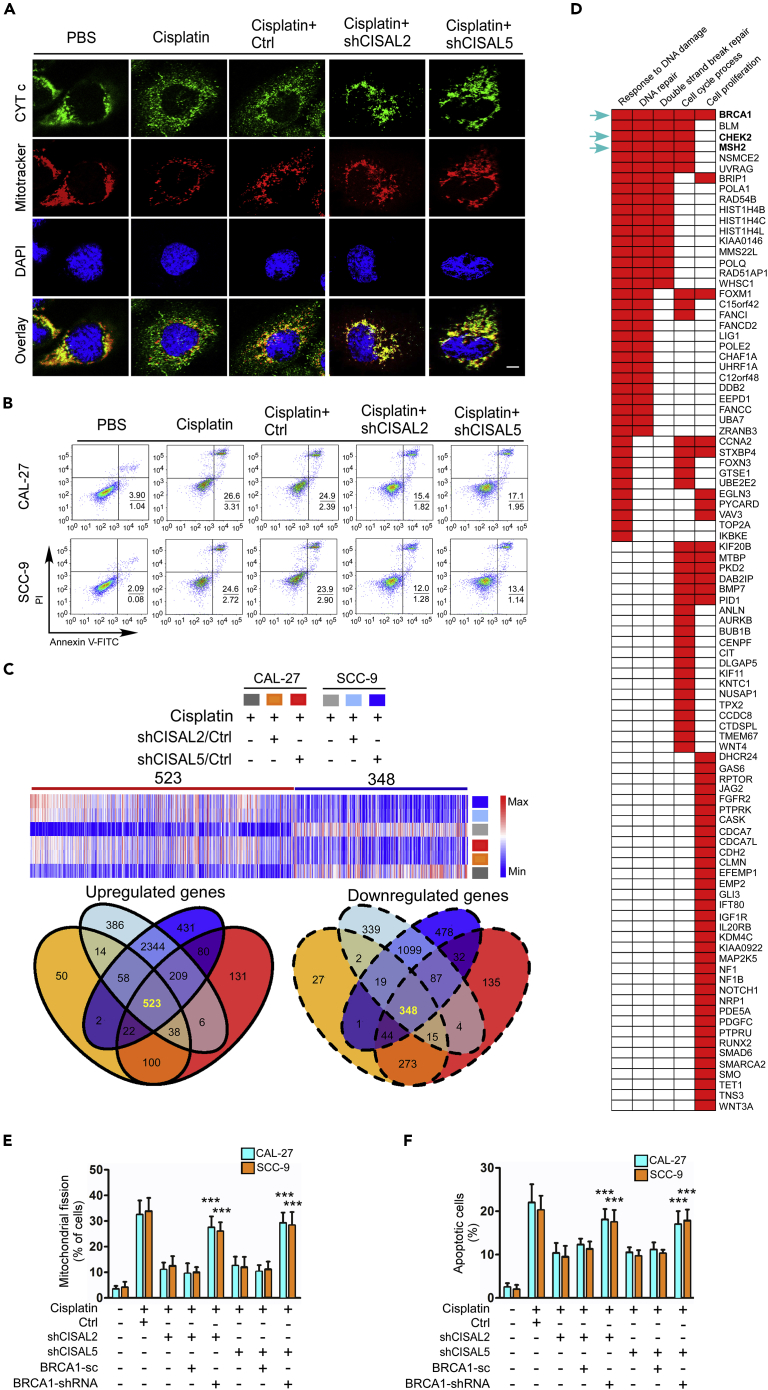
CISAL Promotes Mitochondrial Fission and Cisplatin Sensitivity in TSCC Cells through BRCA1 (A and B) CAL-27 and SCC-9 cells were treated with shRNA against CISAL. Mitochondrial fission and CYT c distribution was detected by staining with MitoTracker Red and antibodies against CYT c, respectively (A); cell apoptosis was detected using flow cytometry (B). (C) CAL-27 and SCC-9 cells stably expressing two different shRNAs against CISAL (shCISAL2 and shCISAL5) were treated with cisplatin and their RNA was extracted and analyzed for mRNA profiling. Heatmap (upper panel) and Venn diagrams (lower panel) depict differentially expressed mRNAs in cisplatin-treated cells stably expressing shCISAL (fold change ≥2.0). Blue to red color gradation is based on the ranking of each condition from minimum (blue) to maximum (red). (D) Gene set enrichment analysis (GSEA) showing five significantly induced pathways related to the genes upregulated in response to CISAL knockdown in both TSCC cell lines under cisplatin treatment. (E and F) The inhibitory effect of CISAL knockdown on mitochondrial fission, analyzed by staining with MitoTracker red (E), and apoptosis using flow cytometry (F), after BRCA1 silencing. ***p<0.001, 2-tailed Student's t test. Data are represented as mean ±SEM.

We tested whether CISAL could regulate mitochondrial fission and cisplatin sensitivity in TSCC cells. CISAL knockdown by shRNA attenuated mitochondrial fission and cell apoptosis upon cisplatin treatment in TSCC cells (Figures 2A, 2B, S3E, and S3F). Moreover, the release of cytochrome c (CYT c) from the intermembrane space of the mitochondria to the cytosol and caspase-3/7 activity were attenuated upon CISAL silencing in TSCC cells under cisplatin treatment (Figures 2A and S3G). In contrast, mitochondrial fission and apoptosis were increased by enhanced CISAL expression (Figures S3H–S3J). Meanwhile, overexpression of CISAL abolished the inhibitory effect of CISAL knockdown on mitochondrial fission and apoptosis, excluding the possibility that the inhibitory effect was affected by off-target effect of CISAL siRNAs (Figures S3K and S3L). Further, we wondered whether the upregulation of CISAL expression is only prevalent in cisplatin-treated cells. As expected, adriamycin (ADR) and camptothecin (CPT) did not have any effect on CISAL expression in two TSCC cells (Figure S3M). Interestingly, TCGA database analysis showed that higher expression of CISAL correlated with better prognosis in multiple types of human cancer, including bladder carcinoma, low-grade glioma, lung adenocarcinoma, ovarian cancer, and pancreatic adenocarcinoma, further supporting the tumor suppressor role of CISAL in human cancer (Figure S4).

### CISAL Regulates Mitochondrial Fission and Cisplatin Sensitivity Through BRCA1

Recent studies have suggested that lncRNAs have regulatory roles in the transcriptional control of protein-coding genes both in *cis* and in *trans*, and the number of known lncRNA functions is growing rapidly (Carpenter et al., 2013). To explore the downstream target genes of lncRNAs involved in regulating mitochondrial fission and cisplatin sensitivity in TSCC cells, we used RNA profiling technology to simultaneously analyze the mRNA expression levels of genes that were differentially regulated by CISAL. In cisplatin-treated cells, silencing of CISAL by two different shRNAs (shCISAL2 and shCISAL5) led to upregulation of 523 genes, whereas 348 genes were downregulated in both the CAL-27 and SCC-9 cell lines (fold change≥2) (Figure 2C). Gene set enrichment analysis (GSEA) (Subramanian et al., 2005) identified five and three significantly activated pathways for upregulated and downregulated genes, respectively (Figures 2D and S5A). Of these genes, BRCA1 was the most prominent in response to DNA damage, DNA repair, and double-strand break repair pathways (Figure 2D). Cisplatin is known to induce proapoptotic effect by damaging DNA (Hu et al., 2016), and BRCA1 plays a crucial role in the DNA damage response (Schrock et al., 2017). Our previous study also revealed that BRCA1 transactivates miR-593-5p expression and downregulates MFF to attenuate cisplatin sensitivity and mitochondrial fission in TSCC cells (Fan et al., 2015b). Thus, we hypothesized that CISAL knockdown attenuates mitochondrial fission and TSCC cell apoptosis upon cisplatin treatment potentially through regulating BRCA1 expression. Indeed, CISAL knockdown led to a significant increase in BRCA1 levels under cisplatin treatment, and downstream genes of BRCA1, including miR-593 and MFF (Fan et al., 2015b), were upregulated and decreased, respectively (Figures S5B–S5D). In contrast, overexpression of CISAL induced a reverse effect (Figures S5E–S5G). These data suggest that BRCA1 is a downstream target gene of CISAL. To confirm the association of CISAL and BRCA1 in regulating mitochondrial fission and cisplatin sensitivity, we used shRNA to knockdown BRCA1 expression and observed that the inhibitory effect of CISAL knockdown on mitochondrial fission and apoptosis under cisplatin treatment was attenuated by BRCA1 silencing (Figures 2E, 2F, S5H, and S5I). Meanwhile, BRCA1 mRNA levels were also confirmed by qRT-PCR (Figure S5J). All together, these data suggest that CISAL mediates mitochondrial fission and cisplatin sensitivity in TSCC cells by regulating BRCA1 expression. Notably, TCGA analysis showed that lower expression of BRCA1 correlates with good prognosis in multiple types of human cancer, including bladder carcinoma, lower-grade glioma, lung adenocarcinoma, pancreatic adenocarcinoma, invasive breast carcinoma, papillary renal cell carcinoma, and head neck squamous cell carcinoma (Figure S6), further supporting the clinical relevance of BRCA1 signaling pathway.

### CISAL Directly Binds to BRCA1 Promoter and Forms RNA–DNA Triplex Structure

**Figure 3:**
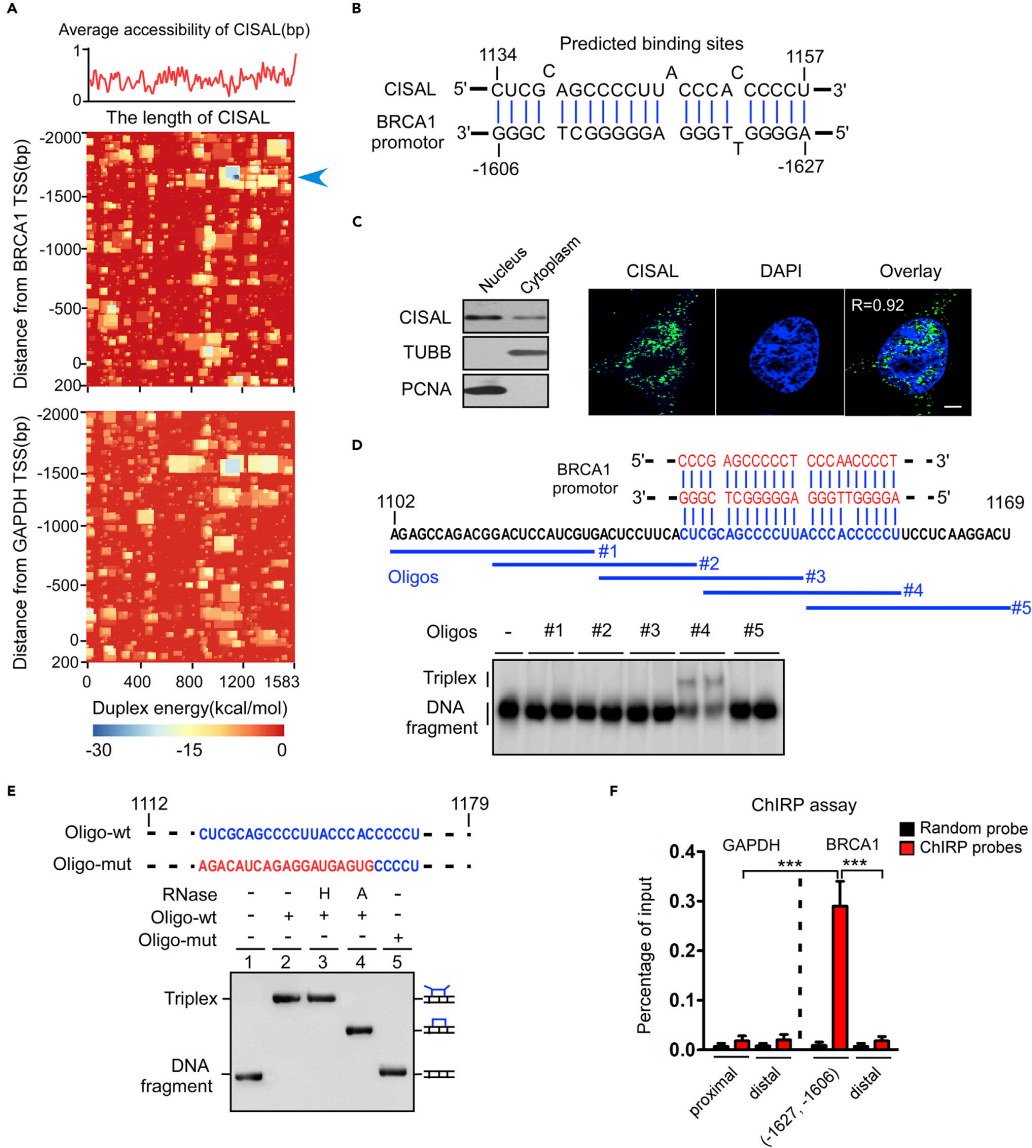
CISAL Forms DNA: RNA Triplex Structure with the BRCA1 Promoter (A) Binding potential between CISAL and BRCA1 or GAPDH promoter regions using IntaRNA. The red curve shows the average probability of single-stranded RNA. Heatmap represents the base-pairing energy for an RNA/RNA duplex model for seed-based regions along the CISAL transcript and 2,000 bp upstream and 200 bp downstream of the TSS of BRCA1 (upper) and GAPDH (lower). (B) Representation of the predicted interaction of CISAL sequence and the promoter DNA region of BRCA1 at the lowest free energy loci. (C) Northern blotting (left panel) and FISH (right panel) revealing that CISAL was located in both the nucleus and cytoplasm but predominantly in the nucleus. Scale bar, 3 μm. (D) Oligo #4 forms a triplex structure with the BRCA1 DNA promoter. The indicated RNA oligos (to #5) were incubated with a biotin-labeled DNA promoter fragment (−1655, −1586) and the formation of DNA: RNA triplexes were monitored by EMSA. The tiled 20 nt RNA oligos (blue) are displayed above. (E) DNA:RNA triplexes are resistant to RNase H digestion. Triplexes (lanes 2–4), formed by incubating a biotin-labeled BRCA1 DNA promoter fragment (−1685, −1566) with 68-nt wild RNA oligos (1112, 1179) (Oligo-wt), were treated with 30 U of RNase H (H) or RNase A (A) and analyzed by EMSA. Lane 5 shows DNA fragments incubated with the mutant RNA oligos (Oligo-mut). (F) ChIRP analysis of CISAL in the regulatory regions of BRCA1(−1627, −1606) but not GAPDH. The cross-linked CAL-27 cell lysates were incubated with biotinylated DNA probes against CISAL, and the binding complexes were recovered using streptavidin-conjugated magnetic beads. qPCR was performed to detect enrichment of the specific regulatory regions associated with CISAL. ***p<0.001, one-way ANOVA followed by Dunnett's tests for multiple comparisons. Data are represented as mean ±SEM.

We next aimed to address the important question of how CISAL targets BRCA1 in *trans*. Study of genomic association showed that lncRNAs can interact with gene promoters by forming RNA–DNA triplex, possibly through Hoogsteen base pairing, to regulate the target gene expression (Mondal et al., 2015). To explore potential lncRNA-binding sites within the BRCA1 promoter, we calculated the binding potential of CISAL to fragments covering 2000 bp upstream and 200 bp downstream of the transcriptional start sites (TSSs) of BRCA1 and GAPDH (control) by IntaRNA. The heatmap revealed a short stretch in the CISAL RNA that was predicted to bind to a complementary region in the BRCA1 promoter at (−1627, −1606), whereas the GAPDH promoter was negative throughout the region (Figures 3A and 3B).

To investigate the interaction of CISAL and BRCA1 regulation, we evaluated their subcellular location. Northern blotting and RNA fluorescence *in situ* hybridization (Kim et al., 2015) showed that substantial amounts of CISAL were mainly visible in the nucleus in CAL-27 cells (Figure 3C), consistent with the results from TSCC tissues (Figure 1E). We also found that localization of CISAL was not significantly changed upon cisplatin treatment or overexpression of CISAL (Figures S7A and S7B). To experimentally examine whether the predicted CISAL matching sequences have the potential to form a triple-stranded structure with the BRCA1 promoter, we incubated biotin-labeled DNA fragments (BRCA1-70, 70 nt) with tiled 20 nt RNA oligonucleotides and analyzed the formation of RNA-DNA triplexes by electrophoretic mobility shift assay (EMSA) (Figure 3D). The oligoribonucleotide sequence (oligo #4) retarded the mobility of the DNA fragment, unlike any other oligos, suggesting that oligo #4 interacts with the DNA, presumably by forming a triplex structure with the predicted sequence. On the other hand, treatment with RNase H did not affect the mobility of the RNA-DNA complex, ruling out the possibility that the shift in electrophoretic mobility is due to the formation of DNA-RNA heteroduplexes (Figure 3E). Again, no triplex formation was observed with mutant oligo (Figure 3E), reinforcing the necessity for a specific sequence between lncRNA and DNA to form triplex structures. We also examined whether CISAL could bind to the chromatin of the regulatory regions of BRCA1 and GAPDH genes by performing a chromatin isolation using RNA purification (ChIRP) assay. The cross-linked CAL-27 cell lysates were incubated with biotinylated DNA probes against CISAL, and the binding complexes were recovered using streptavidin-conjugated magnet beads. Enrichment of the specific regulatory regions of BRCA1, but not of the unrelated region or GAPDH gene, by CISAL was detected (Figure 3F). Moreover, cisplatin treatment enriched CISAL-binding regions at BRCA1 promoter as demonstrated by a ChIRP assay (Figure S7C). Interestingly, lncRNA NBR2 has been fairly well studied (Liu et al., 2016), and CISAL triplex can actually form in the region within the first intron of lncRNA NBR2. Taking that into consideration, we investigated whether regulation of CISAL expression had any effect on lncRNA NBR2 expression level. Our data revealed that lncRNA NBR2 expression level was not affected by CISAL overexpression/knockdown (Figures S7D and S7E). Taken together, these results suggest that CISAL might be recruited to the specific regulatory region of BRCA1 and form triplex structures.

### CISAL Facilitates Transcriptional Repression by Counteracting GABPA Binding with BRCA1 Promoter

We evaluated how the interaction of CISAL and BRCA1 promoter inhibits BRCA1 expression. Recent discoveries demonstrated that the cellular localization of lncRNAs is informative regarding its function, whereas nuclear lncRNAs could plausibly have functions in histone modification (Greco and Condorelli, 2015) or direct transcriptional regulation (Long et al., 2017). Therefore, we first sought to determine whether CISAL regulates BRCA1 via histone methylation or acetylation. ChIP assays showed that CISAL overexpression or silencing had no effect on H3 methylation or H3/H4 acetylation of the promoter of BRCA1 (Figures S8A–S8D). Moreover, inhibitory efficacy of CISAL overexpression on BRCA1 expression was comparable between CAL-27 and SCC-9 cells under the treatment of AZA/TSA or PBS (Figures S8E and S8F). Hence, it is most likely that CISAL-mediated BRCA1 expression is not regulated by the “first hit”—histone modification.

**Figure 4:**
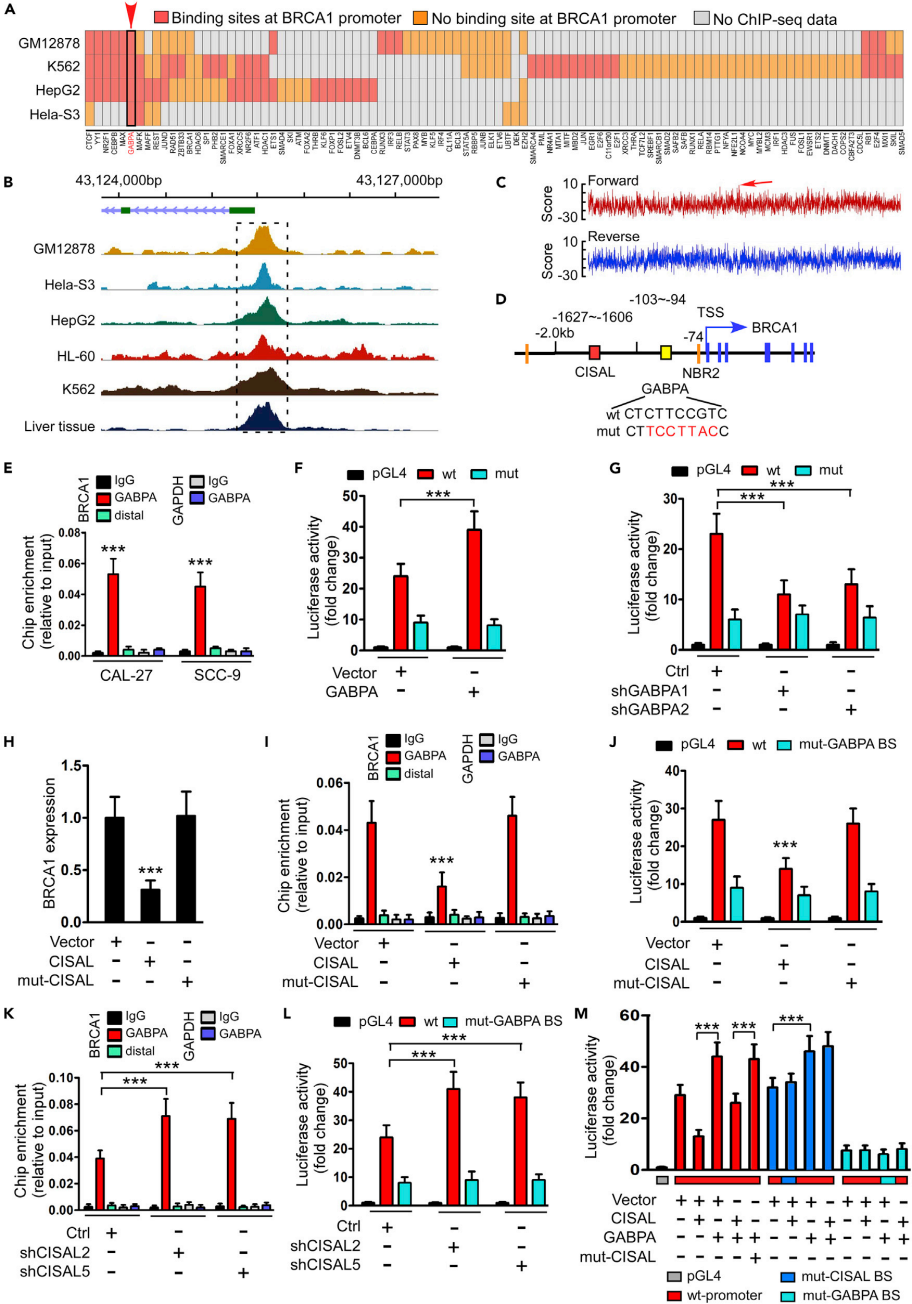
CISAL Represses BRCA1 Transcriptional Activity through Inhibition of GABPA Binding with BRCA1 Promoter (A) Heatmap of selected TFs from Entrez cancer gene list in four commonly used ENCODE cell lines. Cells are sorted based on their RNA-seq data, and the color indicates whether each TF has binding peaks in the BRCA1 promoter. The TFs are hierarchically clustered using Ward's method. (B) Distribution of GABPA occupancy frequencies in BRCA1 promoter in five different cancer cell lines and liver tissue based on ChIP-seq database. The most enriched peaks are highlighted. (C) Motif analysis (motif-counter) showing enriched GABPA motif in BRCA1 promoter and the arrow indicates that the highest score binding sites consistently located in the forward strand, highlighted in panel B. (D) Representation of CISAL and GABPA binding elements in BRCA1 promoter. (E) ChIP-qPCR analysis of the GABPA genomic occupancy in the BRCA1 promoter in CAL-27 and SCC-9 cells as indicated. Immunoprecipitated DNA was measured by real-time PCR with primers to amplify the BRCA1 promoter region, including the distal site, or the GAPDH locus as a negative control region. (F) Luciferase reporter assay demonstrating that GABPA activated BRCA1 promoter activity. CAL-27 cells with stable expression of pGL4.20 empty vector (Vector) and wild-type (wt-GABPA BS) or mutant (mut-GABPA BS) BRCA1-promoter-delivered pGL4.20 vectors were transiently co-transfected with GABPA expressing plasmids and pRL-TK. (G) Luciferase assay demonstrating that GABPA knockdown inhibits BRCA1 promoter activity in CAL-27 cells. (H–J) Overexpression of wild-type CISAL but not the mutant CISAL (mut-CISAL) represses BRCA1 expression (H), GABPA occupancy in BRCA1 promoter (I) and BRCA1 transcriptional activity (J) in CAL-27 cells. (K and L) Knockdown of CISAL increases GABPA occupancy at BRCA1 promoter (K) and BRCA1 promoter activity (L) in CAL-27 cells. (M) Luciferase assay demonstrating that GABPA overexpression attenuates the inhibition of BRCA1 promoter activity, by enhancing expression of CISAL but not mut-CISAL in CAL-27 cells stably transfected with wild-type BRCA1 promoter, whereas overexpression of CISAL demonstrated no effect on BRCA1 promoter activity when transfected with mutant CISAL-binding sites (mut-CISAL BS), and the luciferase signals were similar in groups transfected with mutant GABPA binding sites (mut-GABPA BS). ***p<0.001 by 2-tailed Student's t test (E) or 1-way ANOVA followed by Dunnett's tests for multiple comparisons (F–M). Data are represented as mean ±SEM.

During the last decade, investigation has uncovered that many lncRNAs can actively modulate the DNA-binding activity of their associated TFs by acting as non-DNA binding cofactors (Rapicavoli et al., 2011). We therefore evaluated whether CISAL could interfere with the BRCA1 TFs at the promoter. The ENCODE project (encodeproject.org/ENCODE/) produced numerous ChIP-sequencing (ChIP-seq) datasets that map the genomic locations of TF binding in various types of tissues and cell lines. Cancer-associated TFs can be defined by combining the Entrez cancer gene list (Domazet-Loso and Tautz, 2010) with ENCODE. We screened the human ENCODE database for cancer-associated TFs that interact with BRCA1 promoter in four of commonly used cancer cell lines (GM12878, K562, HepG2, and Hela-S3) and identified dozens of TFs that are associated with the BRCA1 promoters (Figure 4A). Among these TFs, GABPA was most frequently enriched in the common region at BRCA1 promoter in five cancer cell lines and liver tissues (Figure 4B). We further performed de novo motif discovery on GABPA peaks and observed that the 5′–CTCTTCCGTC–3′ (reverse complement: 5′–GACGGAAGAG–3′) motif was highly enriched (Figures 4C and 4D). Interestingly, GABPA has been reported to be a critical activator of BRCA1 expression (Atlas et al., 2000). As expected, we found that GABPA knockdown reduced BRCA1 levels in CAL-27 and SCC-9 cells (Figures S9A and S9B), whereas overexpression of GABPA upregulated BRCA1 levels in both cell lines (Figure S9C). ChIP and luciferase reporter assays identified the transcriptional functionality of GABPA as well (Figures 4E–4G).

We then explored the effect of CISAL on GABPA transcriptional activity. Enforced expression of CISAL but not mut-CISAL (contains a mutant binding site in BRCA1 promoter region) reduced BRCA1 expression (Figure 4H), GABPA/RNA pol II occupancy (Figures 4I, S9D, and S9E), and transcriptional functionality of BRCA1 promoter (Figure 4J), subsequently modulating BRCA1 downstream signaling miR-593-MFF (Figures S9F and S9G). Furthermore, functional assays showed that enforced CISAL, but not mut-CISAL, increased mitochondrial fission and cisplatin sensitivity in CAL-27 and SCC-9 cells (Figure S9H). In contrast, silencing CISAL induced upregulation of GABPA/RNA pol II occupancy (Figures 4K, S9I, and S9J), transcriptional functionality of BRCA1 promoter (Figure 4L), and modification of BRCA1 downstream signaling (Figures S9K and S9L). Luciferase reporter assay further confirmed that enforced GABPA levels attenuated inhibition of BRCA1 transcriptional activity by CISAL overexpression. However, transfection of mutant CISAL-binding sites in BRCA1 promoter abolished the effect of enforced CISAL expression, wherease mutant GABPA-binding sites abrogated the effect of both CISAL and GABPA overexpression on BRCA1 transcriptional activity (Figure 4M). All together, these results show that GABPA is a key transcriptional activator in our cellular system and that CISAL inhibits BRCA1 expression through counteracting GABPA binding at the BRCA1 promoter region.

### CISAL Sequesters GABPA Away from Regulatory Binding at BRCA1 Promoter

**Figure 5:**
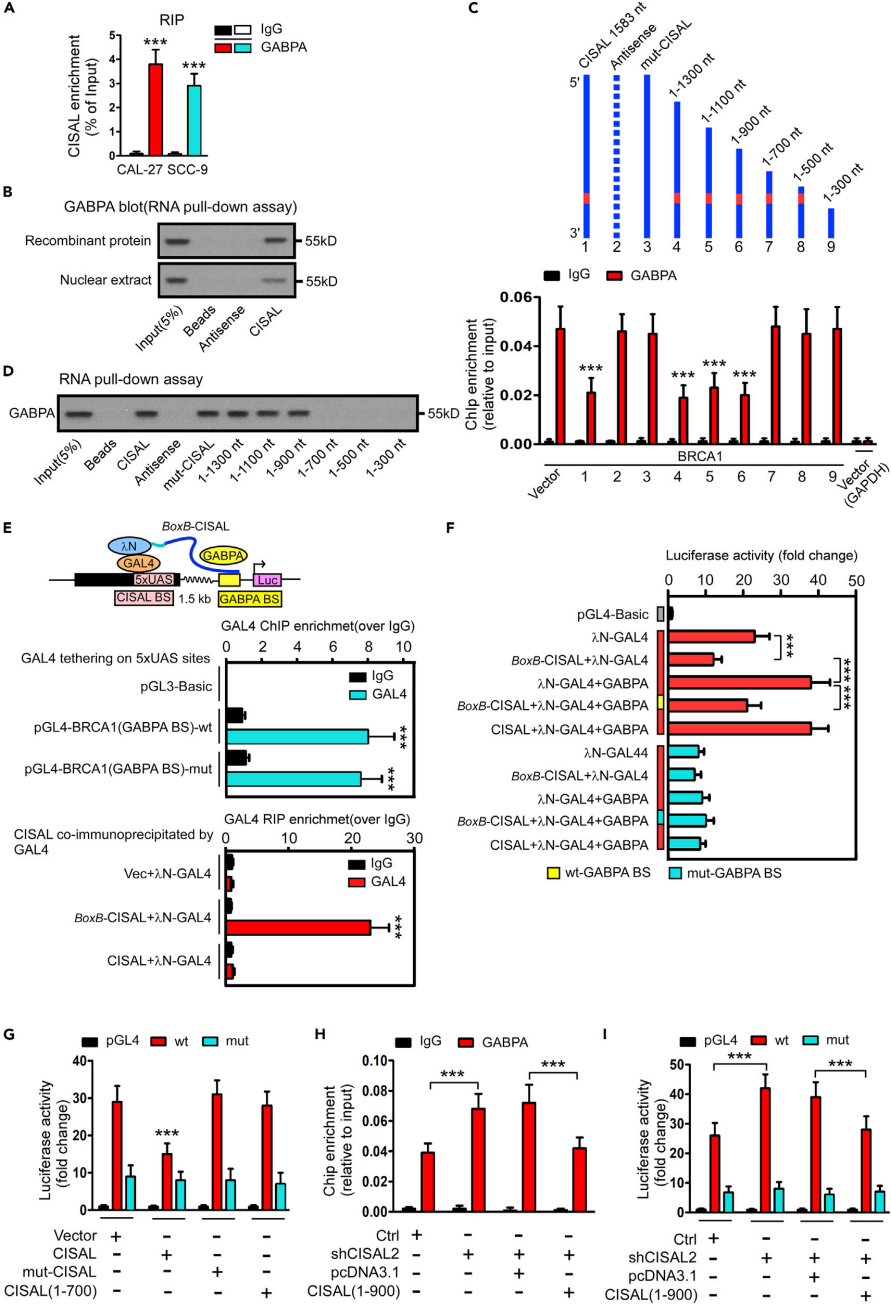
CISAL Sequesters GABPA away from Regulatory Binding Sites at BRCA1 Promoter (A) RT-qPCR analysis of CISAL enrichment by GABPA in the RIP assay in CAL-27 and SCC-9 cells. Normal IgG was used as a nonspecific control. (B) Western blot analysis showing that CISAL associates with GABPA, as indicated by the pull-down assay with *in vitro* translated GABP or nuclear extracts of CAL-27 cells. Antisense CISAL was used as a negative control RNA in the pull-down assay. (C and D) Serial deletions of CISAL were used in ChIP-qPCR analysis (C) and RNA pull-down assays (D) to identify valid length of CISAL, required for physical interaction with GABPA and for sequestering GABPA away from the its downstream binding sites at BRCA1 promoter in CAL-27 cells. Immunoprecipitated DNA was measured by real-time PCR with primers to amplify the BRCA1 promoter region, including the GAPDH locus as a negative control. (E) Schematic diagram of the *x*-tethering system on CISAL, which is upstream of GABPA binding sites in BRCA1 promoter-linked luciferase (Luc). CISAL-binding sequence was substituted with 5xUAS and a chimeric RNA by fusing CISAL to *BoxB* viral RNA *BoxB*-CISAL. N-GAL4 fusion protein tethers *BoxB*-CISAL to the 5xUAS sites. Middle panel indicate efficiency of GAL4 tethering at 5xUAS region by ChIP-qPCR analysis. Lower panel shows that BoxB-CISAL instead of CISAL co-immunoprecipitated by GAL4. (F) Luciferase assay shows *BoxB*-CISAL repressed BRCA1 promoter activity, which was rescued upon enhancement of GABPA expression in CAL-27 cells with wild GABPA-binding sites transfection and using mutant GABPA-binding sites as a negative control. (G) Site-directed mutagenesis of 1–700 nt of CISAL leads to a loss of the effect on BRCA1 promoter activity in CAL-27 cells. (H and I) Forced expression of the truncated CISAL (1–900) abolished the increase of GABPA occupancy (H) and BRCA1 transcriptional activity (I) by silencing endogenous CISAL in CAL-27 cells. ***p< 0.001 by 2-tailed Student's t test (A, C, E, and G–I) or 1-way ANOVA followed by Dunnett's tests for multiple comparisons (F). Data are represented as mean ±SEM.

To determine the precise role of CISAL in interfering with GABPA binding, we first considered CISAL competing GABPA-binding sequence; however, no possible accessibility exists near GABPA-binding region (Figure 3A), and ChIRP assay also identified no occupancy of CSIAL in GABPA target DNA sequence (Figure S9M). We therefore decided to focus on the interaction between CISAL and GABPA, because CISAL was tethered upstream of GABPA-binding sites by DNA-RNA formation (Figure 3) where it may possibly sequester GABPA away from the regulatory region. RNA immunoprecipitation (RIP) assays revealed that CISAL could interact with endogenous GABPA in TSCC cells (Figure 5A). RNA pull-down assay also identified that CISAL could interact with recombinant GABPA and endogenous GABPA in TSCC cells (Figure 5B). To ascertain how CISAL interacted with GABPA, we used serial CISAL deletion analysis (Figures 5C and 5D) and ChIP-qPCR, which revealed that CISAL deletion to 900nt from its 5′-end preserved its ability to block GABPA binding, whereas the truncated 700nt abrogated such effect (Figure 5C). Consistently, RNA pull-down assay indicated that CISAL deletion to 900nt presented affinity with endogenous GABPA (Figure 5D). Furthermore, we engineered an allele of CISAL that can be artificially recruited to upstream binding sites of GABPA in GAL4-*BoxB*-tethering-based reporter assay (Li et al., 2013). Addition of *BoxB* RNA element to CISAL (*BoxB*-CISAL) allowed the fusion transcript to be recruited by the RNA-binding domain of λN protein fused with the GAL4 DNA-binding domain (λN–GAL4) when CISAL-binding sequence was deleted and substituted with 5xUAS sites (Figure 5E). We confirmed that GAL4 could be tethered at 5xUAS region and *BoxB*-CISAL co-immunoprecipitated by GAL4 (Figure 5E). Luciferase assay indicated recruitment of CISAL at 5xUAS site, significantly repressing transcription, but enforced overexpression of GABPA attenuated the inhibition if the promoter included wild GABPA-binding sites. However, the mutant GABPA sequence abolished the effect of both *BoxB*-CISAL and GABPA on transcriptional activity (Figure 5F). These data suggest that CISAL is specifically tethered upstream of GABPA-targeted DNA sequence, subsequently sequestering GABPA away from its regulatory binding sites at BRCA1 promoter and inhibiting BRCA1 transcription.

Next, we confirmed the role of truncated CISAL in modulating BRCA1 signaling pathway, mitochondrial fission, and cisplatin sensitivity in TSCC cells. CISAL (1-700) was unable to inhibit BRCA1 expression (Figure S10A) and transcriptional activity (Figures 5G, S10B, and S10C), or BRCA1 downstream signaling (Figure S10D, E), consequently losing the ability to boost mitochondrial fission and cisplatin sensitivity (Figure S10F). In contrast, overexpression of truncated CISAL (1-900) in CISAL-silenced cancer cells restored the function of CISAL related to BRCA1 levels (Figure S10G), GABPA/RNA pol II occupancy (Figures 5H, S10H, and S10I), BRCA1 transcriptional activity (Figure 5I), expression of downstream genes (Figures S10J and S10K), as well as mitochondrial fission and cisplatin sensitivity (Figure S10L).

### CISAL Regulates Cisplatin Chemosensitivity in TSCC *In Vivo*

**Figure 6:**
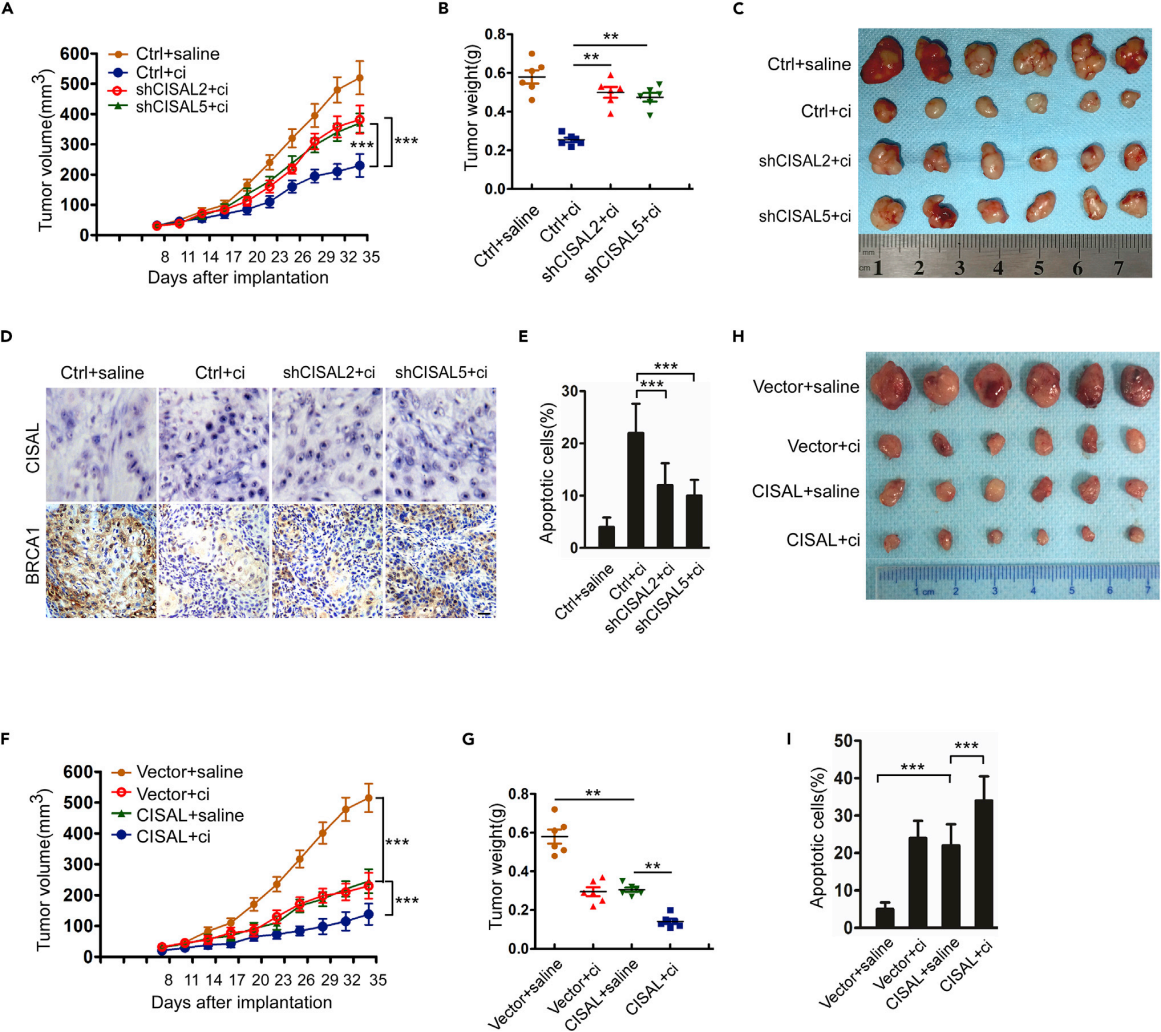
CISAL Regulates Apoptosis and Cisplatin Sensitivity in CAL-27 Cell Xenografts *In Vivo* (A) BALB/c nude mice bearing xenografts of CAL-27 cells with stable knockdown of CISAL or negative controls (Ctrl) were treated with saline or cisplatin (n = 6 per group) and tumor growth was monitored. Results are expressed as the mean ± SEM. (B) Tumor weight for each group. (C) Photomicrographs of tumors from each group at day 35. (D) CISAL knockdown decreases CISAL expression but upregulates BRCA1 expression in CAL-27 cell xenografts upon treatment with cisplatin. CISAL and BRCA1 expression was detected by ISH and IHC, respectively in tissues from different treated groups. (E) TUNEL assays showed that apoptosis in response to cisplatin was attenuated by CISAL knockdown. (F–I) BALB/c nude mice bearing xenografts of CAL-27 cells with stable CISAL expression or control vector were treated with saline or cisplatin (n = 6 per group) and tumor was monitored over time (F); tumor weight (G) for each group, photomicrographs of tumors (H), and apoptotic dells (I) from each group at day 35. ***p< 0.001, 2-way ANOVA followed by Bonferroni's post-test (A and F); **p<0.01 and ***p<0.001, 1-way ANOVA followed by Dunnett's tests for multiple comparisons (B, E, G, and I); scale bar, 20 μm. Data are represented as mean ±SEM.

To further validate the relationship between CISAL and BRCA1 in the regulation of cisplatin sensitivity, we established TSCC xenografts *in vivo*. CISAL knockdown led to a significant increase in CAL-27 tumor growth in the presence of cisplatin (Figures 6A–6C). CISAL expression was downregulated in the CISAL silencing group, whereas BRCA1 expression was increased (Figures 6D and S11A–S11C). We also analyzed BRCA1 downstream genes (Fan et al., 2015b) and found that the expression of miR-593 was increased in tumors with CISAL knockdown, whereas MFF expression was decreased (Figures S11C andS11D). Apoptosis was also attenuated upon CISAL knockdown under cisplatin treatment (Figures 6E and S11E). On the other hand, PCNA expression was not found to be significantly different in each group (Figure S11C), indicating that the influence of CISAL was not secondary to impaired proliferation. In contrast, overexpression of CISAL inhibited tumor growth and enhanced cisplatin sensitivity (Figures 6F–6H), whereas apoptotic tumor cells, CISAL, BRCA1, miR-593-5p, and MFF expression (Figures 6I and S12A–S12E), were also detected, supporting the idea that enforced CISAL expression inhibits BRCA1 transcription *in vivo*. These results suggest that CISAL regulates cisplatin sensitivity and apoptosis in TSCC cells by directly modulating BRCA1 transcription *in vivo*.

### High CISAL and Low BRCA1 Expression Are Associated with Favorable Neoadjuvant Chemosensitivity and Prognosis of TSCC Patients

**Figure 7:**
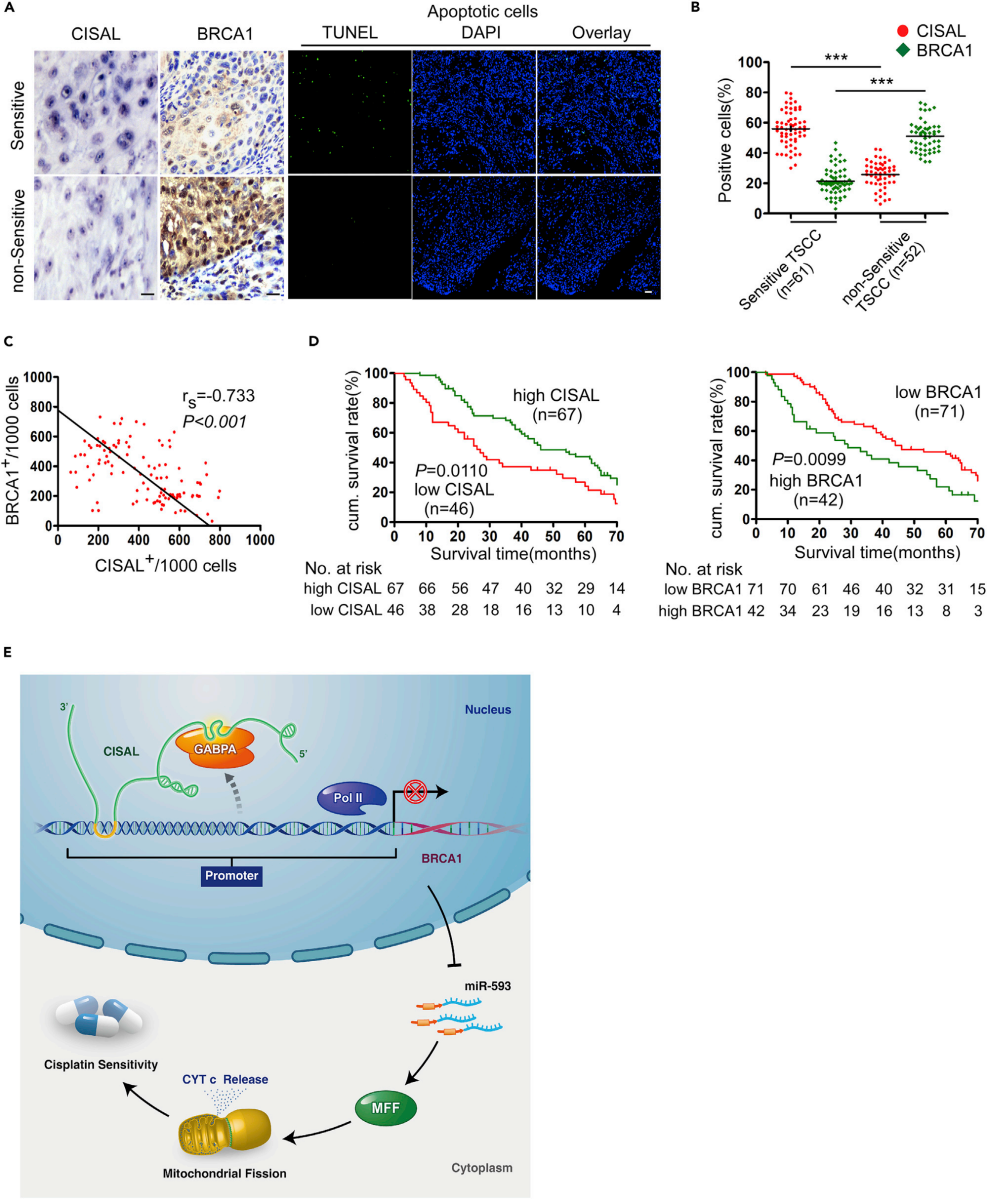
High CISAL and Low BRCA1 Expression Correlates with Favorable Neoadjuvant Chemosensitivity and Prognosis in TSCC Patients (A) CISAL and BRCA1 expression and apoptosis were compared in chemosensitive and nonsensitive TSCC tumor samples. CISAL and BRCA1 expression was analyzed by ISH and IHC (×200), respectively; apoptosis was detected using TUNEL assays; scale bar, 20 μm. (B) Quantification of CISAL and BRCA1 expression in chemosensitive and nonsensitive TSCC tumors; ***p<0.001, 2-tailed Student's t test. (C) Associations of CISAL and BRCA1 expression in TSCC analyzed by Spearman rank order correlation. (D) Kaplan-Meier survival curves for TSCC patients were plotted for CISAL and BRCA1 expression, and survival differences were analyzed using a logrank test. (E) Schematic representation of our proposed model of direct interaction of CISAL with BRCA1 promoter to sequester the downstream GABPA away from transcriptional regulatory binding sites, silencing BRCA1 transcription, subsequently upregulating mitochondrial fission and cisplatin sensitivity in carcinoma cells.

To evaluate the clinical relevance of CISAL and BRCA1 expression, we performed a retrospective analysis of TSCC samples (TSCCs) from 113 patients treated with platinum-based neoadjuvant chemotherapies. *In situ* hybridization (ISH) and immunohistochemical staining demonstrated that CISAL expression was higher, whereas BRCA1 expression was lower in chemosensitive TSCCs, as compared with resistant tumors (Figure 7A). A significant difference in the expression profile of chemosensitive and chemoresistant TSCCs were determined by the percentage of positive cells (Figure 7B). Consequently, cisplatin-sensitive TSCCs exhibited a higher percentage of apoptotic cells (Figure 7A). Additionally, a Spearman order correlation analysis showed that CISAL levels were reversely correlated with those of BRCA1 in TSCCs (rs = −0.733, p<0.001) (Figure 7C). Notably, TCGA analysis also found an inverse correlation in CISAL and BRCA1 expression in bladder carcinoma (Figure S13).

Next, we analyzed the association of CISAL and BRCA1 expression with the clinicopathological status of TSCC patients (Table S3). No significant correlation was observed between CISAL or BRCA1 expression and sex, age, lymph node status, or clinical stage. However, CISAL and BRCA1 expression were significantly associated with cisplatin sensitivity. Moreover, we evaluated the correlation between CISAL and BRCA1 expression and patient overall survival (OS). The cumulative survival rate at 60 months was 43.28% and 43.66% in patients with high CISAL and low BRCA1 expression, respectively; however, the corresponding rate was only 21.74% and 19.05% in those with low CISAL and high BRCA1 expression, respectively (Table S3). A univariate Cox regression analysis indicated that TSCC patients with high CISAL expression level or low BRCA1 levels had longer OS (Table 1 and Figure 7D). Furthermore, a multivariate Cox regression analysis revealed that high CISAL expression and low BRCA1 expression are independent prognostic factors for better OS in patients with TSCC (Table 1). All together, our data suggest that CISAL and its direct target BRCA1 correlate with neoadjuvant chemosensitivity and patient OS with TSCC, whereas CISAL play an important role as DNA binding cofactor by directly interacting with TF, rather than competing in binding with TF or the decoy mechanism (Figure 7E).

**Table 1:** Univariate and Multivariate Analysis of Factors Associated with Overall Survival of Patients with TSCC

Variable	Cases Number	HR (95% CI)	P
**Univariate Analysis**			

Sex Male vs female	62/51	1.107(0.604–2.030)	0.604
Age(years) <50 vs ≥50	37/76	1.186(0.739–1.904)	0.593
Node metastasis N0 vs N+	47/66	1.579(1.169–2.132)	0.023
Clinical stage III VS IV	70/43	2.040(1.368–3.041)	<0.001
Cisplatin Sensitive vs non-sensitive	61/52	0.708(0.509–0.986)	0.046
CISAL Low vs high	46/67	1.687(1.207–2.357)	0.011
BRCA1 Low vs high	71/42	1.716(1.207–2.439)	0.009

**Multivariate Analysis**			

Node metastasis N0 vs N+	47/66	1.686(1.328–2.140)	0.014
Clinical stage III VS IV	70/43	2.119(1.374–3.269)	<0.001
Cisplatin Sensitive vs non-sensitive	61/52	0.667(0.496–0.896)	0.032
CISAL Low vs high	46/67	1.820(1.306–2.537)	0.002
BRCA1 Low vs high	71/42	1.862(1.269–2.731)	<0.001

## Discussion

In this study, we show that the lncRNA CISAL regulates mitochondrial fission and cisplatin sensitivity through BRCA1 signaling. In exploring the mechanism by which BRCA1 transcription is inhibited, we found that CISAL can be specifically tethered at BRCA1 promoter by formation of an RNA–DNA triplex structure, which subsequently sequesters BRCA1 TF-GABPA away from its binding to regulatory DNA target. Importantly, we show that CISAL-BRCA1 expression is associated with patients' survival in multiple types of human cancer and TSCC patients' neoadjuvant chemosensitivity. These results provide mechanistic and translational insights into neoadjuvant chemosensitivity and suggest that targeting CISAL-BRCA1 signaling pathway could be used for predicting or improving neoadjuvant chemosensitivity.

Numerous studies strongly suggest that lncRNAs in “gene desert” regions play significant roles in tumor occurrence and development and could be used as a biomarker (Bonasio and Shiekhattar, 2014, Vance and Ponting, 2014). LncRNAs were initially described as regulators of chromatin organization and gene expression (Engreitz et al., 2013, Giovarelli et al., 2014). LncRNAs can interface with the genome at the sequence level and fold into tertiary structures capable of specific interactions with proteins; therefore, they can regulate gene expression at different levels. In fact, several studies have uncovered many specific examples and general classes of lncRNAs that repress or activate transcription (Bonasio and Shiekhattar, 2014). Indeed, the direct interaction of lncRNAs and TFs to inhibit transcription activities is rarely reported (Long et al., 2017).

In an effort to understand how lncRNAs target the genome, computational approaches are being used to predict the interaction of lncRNAs and chromatin or DNA by (1) suggesting candidate lncRNA-associated TFs according to the enrichments of their binding motifs; (2) proposing the involvement of lncRNA in transcriptional enhancement or repression from enrichments of relevant chromatin markers; and (3) identifying near-complementary DNA sequences within lncRNA-associated regions that might indicate direct RNA–DNA triplex formation (Buske et al., 2012, Vance et al., 2014). Indeed, we first calculated the binding potential and found a complementary sequence between the CISAL and BRCA1 promoter before further experimental validation. The genomic associations observed between lncRNAs and chromatin could be accomplished through direct base pairing between RNA and DNA sequences to form RNA-DNA triplexes, possibly through Hoogsteen base pairing (Mondal et al., 2015). Based on the bioinformatics analysis and rigorously controlled experiments, we identified that CISAL formed RNA–DNA triplex with BRCA1 promoter.

Understandably, nuclear lncRNAs mainly regulate transcription activity through histone modification or direct/indirect interaction with TFs. We observed no association between CISAL and histone modification; however, we found a direct interaction between CISAL and GABPA. Broadly, lncRNAs exercise many roles including recruiters, decoys, stimuli, scaffolds, or some combinations thereof (Long et al., 2017). For the repression of transcription, lncRNA could plausibly act as a“sponge” or “decoy,” binding and activating TF and preventing it from interacting with its DNA/RNA target by binding functionally and inactivating the protein (Kino et al., 2010). LncRNAs may also compete with TFs for DNA-binding sites (Pfingsten et al., 2012) or by determining cocrystal structures of the protein-DNA and protein-lncRNA complexes (Hudson and Ortlund, 2014). Interestingly, we found that only the tethered CISAL at BRCA1 promoter sequestered GABPA away from binding to BRCA1 promoter, because mut-CISAL lost its binding affinity to BRCA1 promoter, thereby disabling transcription repression and even reserving the interaction with GABPA. Therefore, our study proposes a model where lncRNAs specifically regulate gene transcription through tethering lncRNAs at promoter region by RNA–DNA triplex, directly interacting with TF. However, we cannot rule out the possibility that some of the DNA/RNA binding elements are indirectly involved in this procedure.

Emerging data suggest that abnormal mitochondrial morphology may be relevant to various aspects of disease and apoptosis (Trotta and Chipuk, 2017). We have also first revealed the important role of mitochondrial fission in cisplatin sensitivity (Fan et al., 2015a, Fan et al., 2015b). Until now, whether lncRNA is involved in the regulation of cisplatin chemosensitivity through mitochondrial dynamics remains unclear. Our present work indicates that CISAL can regulate mitochondrial fission and cisplatin chemosensitivity through a BRCA1-dependent signaling axis. This work sheds new light to the understanding of mitochondrial fission and chemosensitivity. BRCA1 also plays a pivotal role in DNA repair (Suberbielle et al., 2015). Whether the CISAL/BRCA1 axis mediates the DNA repair pathway requires further investigation. Importantly, our study provides mechanistic and translational insights of CISAL in neoadjuvant chemosensitivity. Regarding the prediction of chemosensitivity, the basal expression of CISAL and the induction of CISAL expression by neoadjuvant chemotherapy had to be detected. Our data demonstrate that higher expression of CISAL was associated with neoadjuvant chemosensitivity. CISAL-BRCA1 axis is not only associated with TSCC patients' neoadjuvant chemosensitivity and OS but is also correlated with OS in multiple types of human cancer, based on TCGA analysis, providing a base for future studies to evaluate the role of CISAL in other types of cancers.

In summary, we are beginning to achieve a full understanding of the molecular mechanism responsible for lncRNA-mediated regulation of transcription. Our study proposes a model where the lncRNA CISAL regulates TSCC mitochondrial fission and cisplatin-based neoadjuvant chemosensitivity, by tethering at BRCA1 promoter and sequestering downstream BRCA1 TF-GABPA away from regulatory binding region, thereby inhibiting BRCA1 transcription and its downstream signaling pathway. Moreover, TCGA analysis revealed that CISAL-BRCA1 axis is associated with OS in multiple types of cancers, suggesting that CISAL-BRCA1 axis could be used as a target to predict or improve neoadjuvant chemosensitivity and patients' overall survival.

### Limitations of the Study

In this study, we demonstrated that CISAL directly binds the BRCA1 promoter and forms an RNA-DNA triplex structure, sequestering BRCA1 transcription factor, GABPA, away from the downstream regulatory binding region, rather than current functionality of lncRNAs in transcriptional regulatory programs, such as competing the binding sites or playing as the decoy/sponge. It is plausible that CISAL plays important roles in 3D chromatin structure formation. Although the short-range chromatin interactions around 2kb cannot be detected by current technology, future studies evaluating CISAL and long-range interactions between the BRCA1 promoter and its enhancers would likely yield deeper mechanistic insight into the regulation of CISAL-BRCA1 signaling pathway. In addition, taking into consideration the CISAL distribution throughout the nucleoplasm and cytoplasm, further studies are needed to identify additional binding partners and functional properties of CISAL.

## Methods

All methods can be found in the accompanying Transparent Methods supplemental file.

## Supplemental Information

Document S1. Transparent Methods, Figures S1–S13, and Tables S1–S5
