# Supplementary material for: lncRNA CISAL Inhibits BRCA1 Transcription by Forming a Tertiary Structure at Its Promoter
Source: iScience. 2020 Jan 11;23(2):100835. doi: 10.1016/j.isci.2020.100835 (PMC7033639; doi:10.1016/j.isci.2020.100835)
Supplement: Document S1. Transparent Methods, Figures S1–S13, and Tables S1–S5 [file mmc1.pdf]

iScience, Volume *23*

Supplemental Information

lncRNA CISAL Inhibits BRCA1 Transcription by Forming a Tertiary Structure at Its Promoter

Song Fan, Tian Tian, Xiaobin Lv, Xinyuan Lei, Zhaohui Yang, Mo Liu, Faya Liang, Shunrong Li, Xiaofeng Lin, Zhaoyu Lin, Shule Xie, Bowen Li, Weixiong Chen, Guokai Pan, Xinyu Lin, Zhanpeng Ou, Yin Zhang, Yu Peng, Liping Xiao, Lizao Zhang, Sheng Sun, Hanqing Zhang, Sigeng Lin, Qunxing Li, Binghui Zeng, Filippos Kontos, Yi Ruan, Soldano Ferrone, Dechen Lin, Bakhos A. Tannous, and Jinsong Li

Supplemental Figures

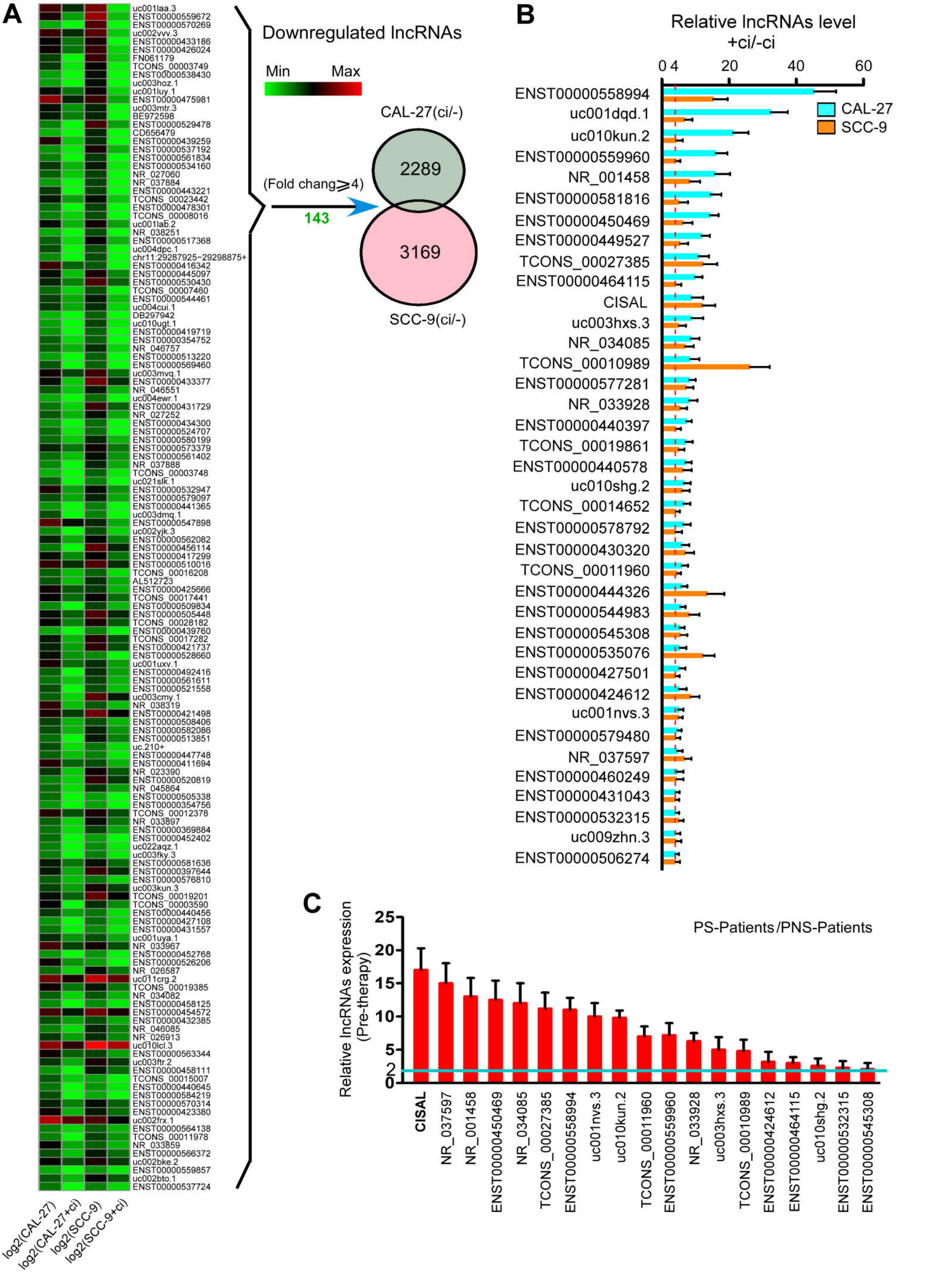

Figure S1. LncRNA profile and validation in TSCC cells and tumors. (A) Heat map showing 143 downregulated lncRNAs in two TSCC cell lines treated with cisplatin compared to untreated control. The

relative lncRNA expression is depicted according to the color scale. Green to red color gradation is based on the ranking of each condition from minimum (green) to maximum (red). (B) 38 upregulated lncRNAs were identified by qRT-PCR in both CAL-27 and SCC-9 cells treated with cisplatin compared to control. (C) Among 38 upregulated lncRNAs, 19 lncRNAs were validated to be increased in chemosensitive (PS) patients before neoadjuvant chemotherapy comparing to chemoresistant (PNS) patients by qRT-PCR. Related to Figure 1.

A

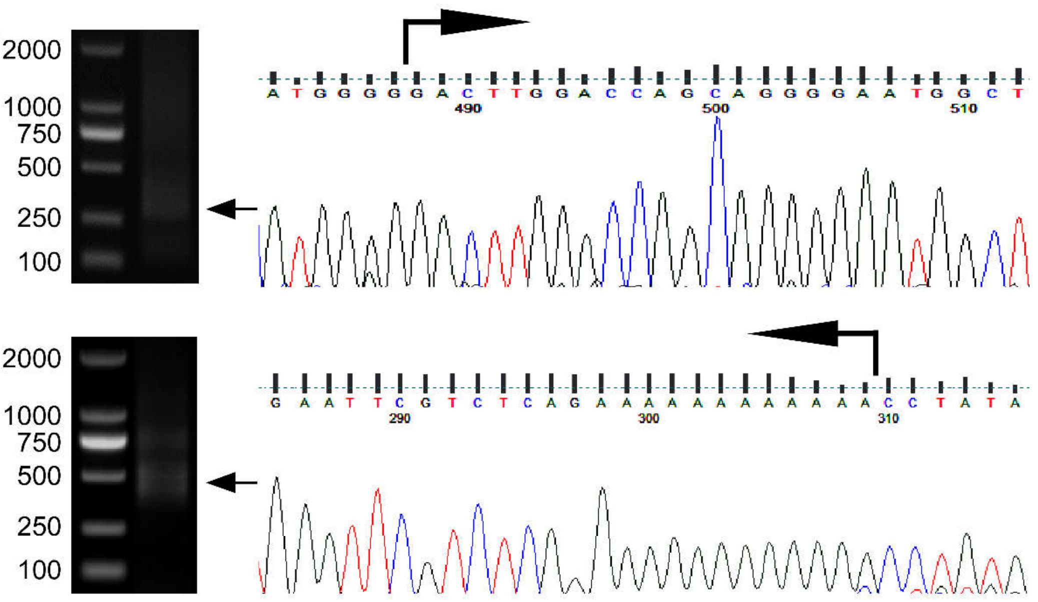

B

Label	Strand	Frame	Start	Stop	Length (nt | aa)
ORF9	-	3	549	229	321 | 106
ORF5	+	3	810	1028	219 | 72
ORF8	-	2	526	371	156 | 51
ORF1	+	1	88	237	150 | 49
ORF7	-	1	398	270	129 | 42
ORF4	+	3	456	563	108 | 35
ORF2	+	1	541	633	93 | 30
ORF3	+	1	754	834	81 | 26
**ORF6**	**-**	**1**	**728**	**648**	**81 | 26**

C

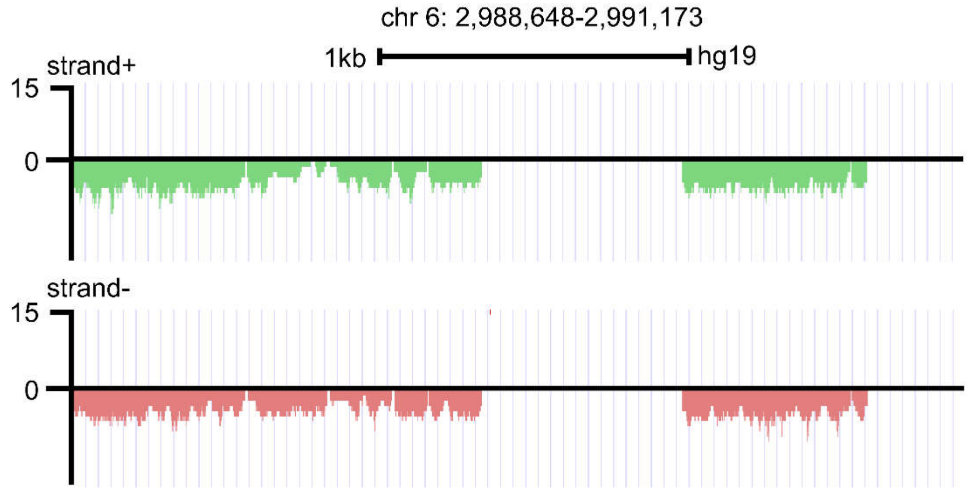

Figure S2. RACE assay of CISAL. (A) 5’ RACE and 3’ RACE of CISAL. Left: gel electrophoresis of nested PCR products from 5' RACE and 3' RACE. The arrow on the right indicates the major PCR product. Right: PCR product sequencing reveals the boundary between the universal anchor primer and CISAL sequences. The vertical line indicates a putative transcriptional start site or a putative transcriptional end site. Arrows indicate transcriptional directions. (B) Prediction of putative proteins encoded by CISAL using ORF Finder (https://www.ncbi.nlm.nih.gov/orffinder). (C) The codon substitution frequency (CSF) scores of CISAL. Related to Figure 1.

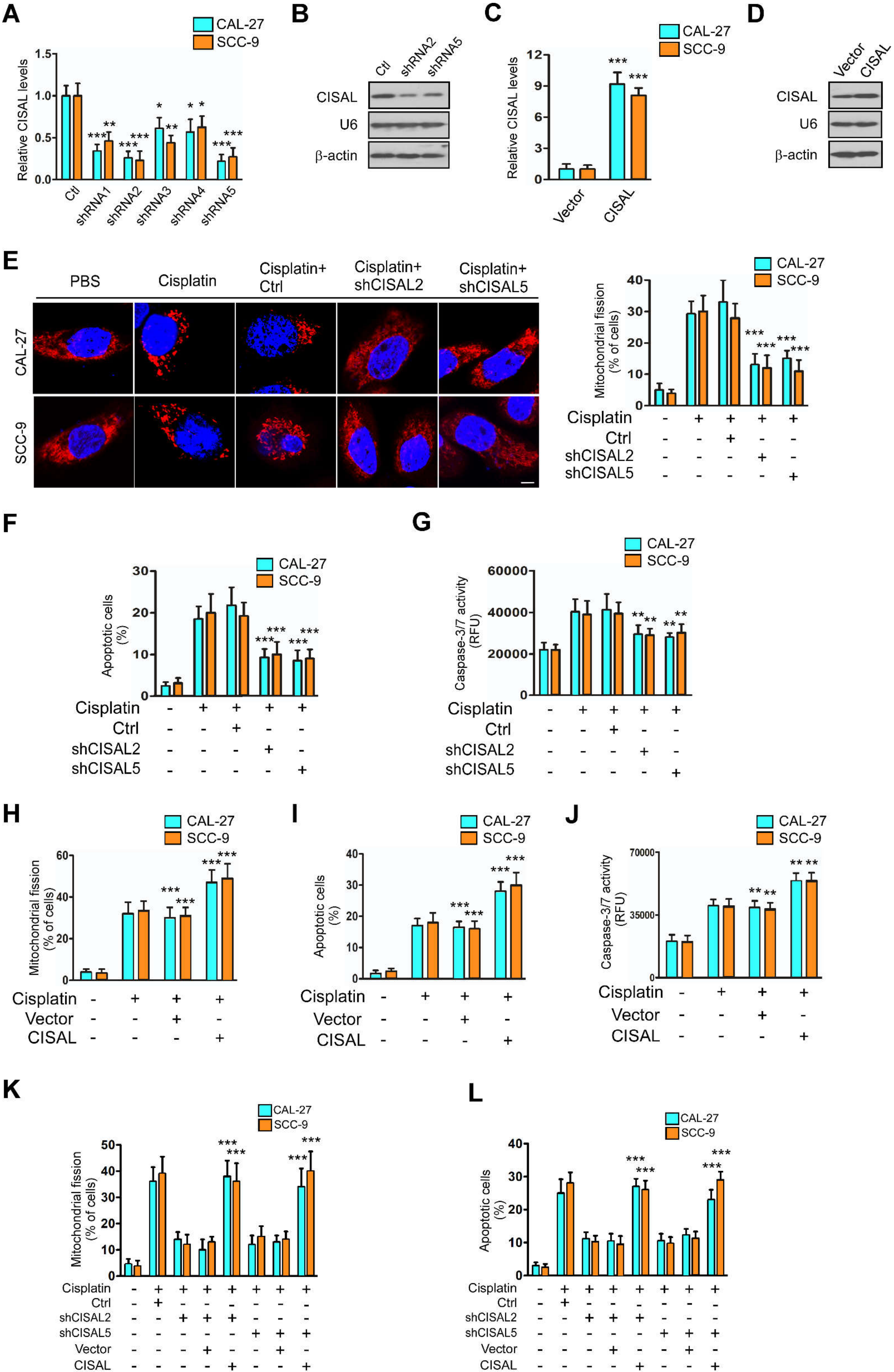
A
CAL-27
SCC-9
Relative CISAL levels
1.5
1.0
0.5
0.0
Ctl
shRNA1
shRNA2
shRNA3
shRNA4
shRNA5
B
Ctl
shRNA2
shRNA5
CISAL
U6
β-actin
C
CAL-27
SCC-9
Relative CISAL levels
12
9
6
3
0
Vector
CISAL
D
Vector
CISAL
CISAL
U6
β-actin
E
PBS
Cisplatin
Cisplatin+ Ctrl
Cisplatin+ shCISAL2
Cisplatin+ shCISAL5
CAL-27
SCC-9
Mitochondrial fission (% of cells)
40
30
20
10
0
Cisplatin
Ctrl
shCISAL2
shCISAL5
F
Apoptotic cells (%)
30
20
10
0
Cisplatin
Ctrl
shCISAL2
shCISAL5
G
Caspase-3/7 activity (RFU)
60000
40000
20000
0
Cisplatin
Ctrl
shCISAL2
shCISAL5
H
Mitochondrial fission (% of cells)
60
40
20
0
Cisplatin
Vector
CISAL
I
Apoptotic cells (%)
40
30
20
10
0
Cisplatin
Vector
CISAL
J
Caspase-3/7 activity (RFU)
70000
35000
0
Cisplatin
Vector
CISAL
K
Mitochondrial fission (% of cells)
50
40
30
20
10
0
Cisplatin
Ctrl
shCISAL2
shCISAL5
Vector
CISAL
L
Apoptotic cells (%)
40
30
20
10
0
Cisplatin
Ctrl
shCISAL2
shCISAL5
Vector
CISAL

Figure S3. Regulation of CISAL expression in TSCC cells. (A) Knockdown efficiency of CISAL by different shRNAs in CAL-27 and SCC-9 cells. (B) Northern blotting demonstrates that CISAL probes specifically detect CISAL in CAL-27 cells. (C) CISAL expression in CAL-27 and SCC-9 cells with stable expression of CISAL. (D) Northern blotting revealed that overexpression of CISAL in CAL-27 cells resulted in a specific increase of CISAL levels. (E-G) CAL-27 and SCC-9 cells were treated with shRNA against CISAL. Mitochondrial fission was detected by staining with MitoTracker Red (left panel) and quantified (right) (E); cell apoptosis was detected using flow cytometry (F), and caspase-3/7 activity assays (G). (H-J) CISAL was overexpressed in CAL-27 and SCC-9 cells and mitochondrial fission (H), cell apoptosis (I) and caspase 3/7 activity (J) was detected. (K and L) The inhibitory effect of CISAL knockdown on mitochondrial fission (MitoTracker red) and apoptosis (flow cytometry) was abolished by CISAL overexpression. (M) CAL-27 and SCC-9 cells were treated with 2×10^{-6} M adriamycin (ADR) (Sigma, USA) or 15×10^{-6} M camptothecin(CPT) (Sigma, USA) for 24 hours to detect CISAL expression. **P*<0.05, ***P*<0.01 and ****P*<0.001 versus control, 2-tailed Student's t test; scale bar, 3 μm. Data are represented as mean ±SEM. Related to Figure 2.

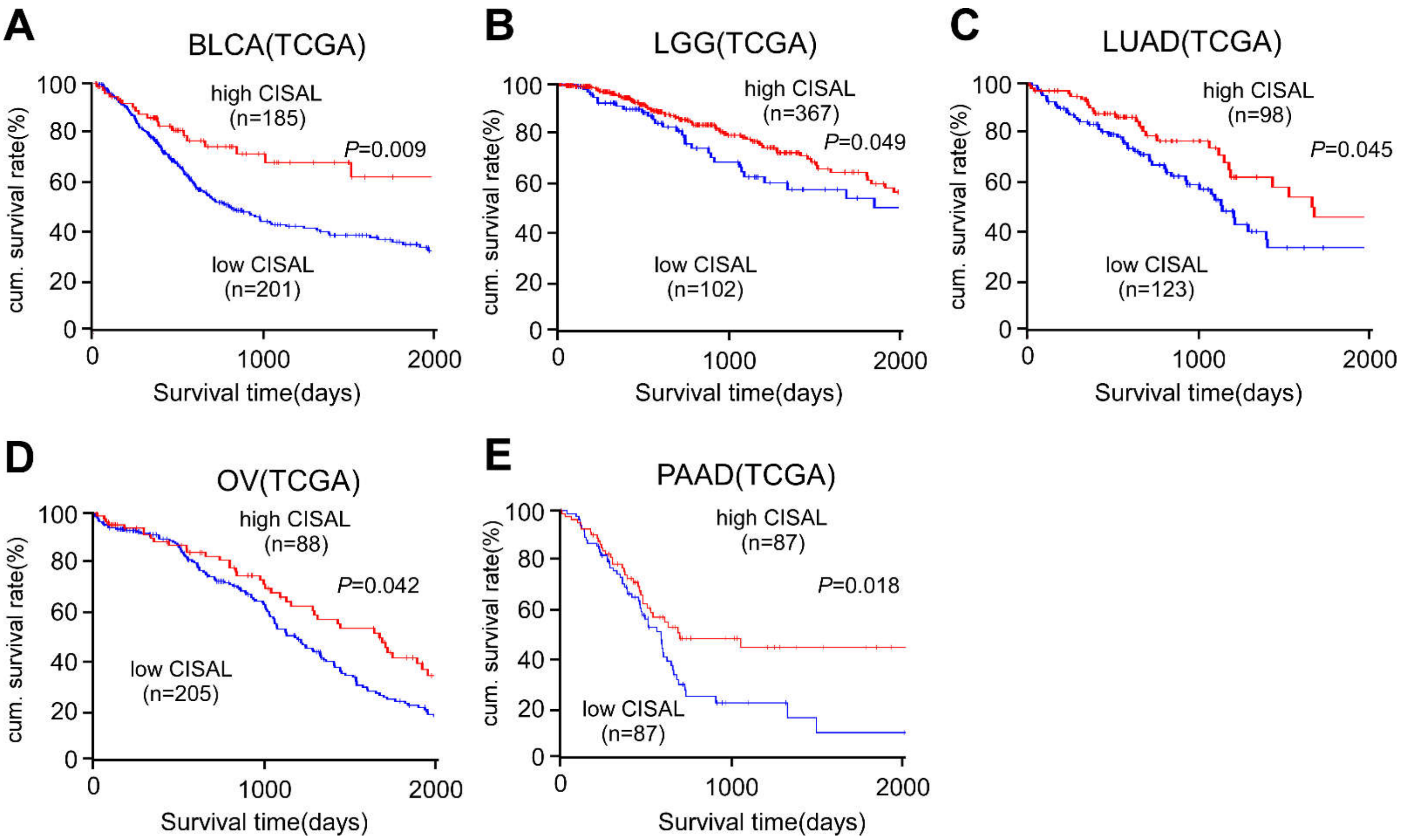

Figure S4. (A-E) TCGA data analysis showing higher CISAL expression is associated with better overall survival in multiple types of cancers including bladder carcinoma (BLCA) (A), low grade gliomas (LGG) (B), lung adenocarcinoma (LUAD) (C), ovarian cancer (OV) (D), and pancreatic adenocarcinoma (PAAD) (E). Related to Figure 2.

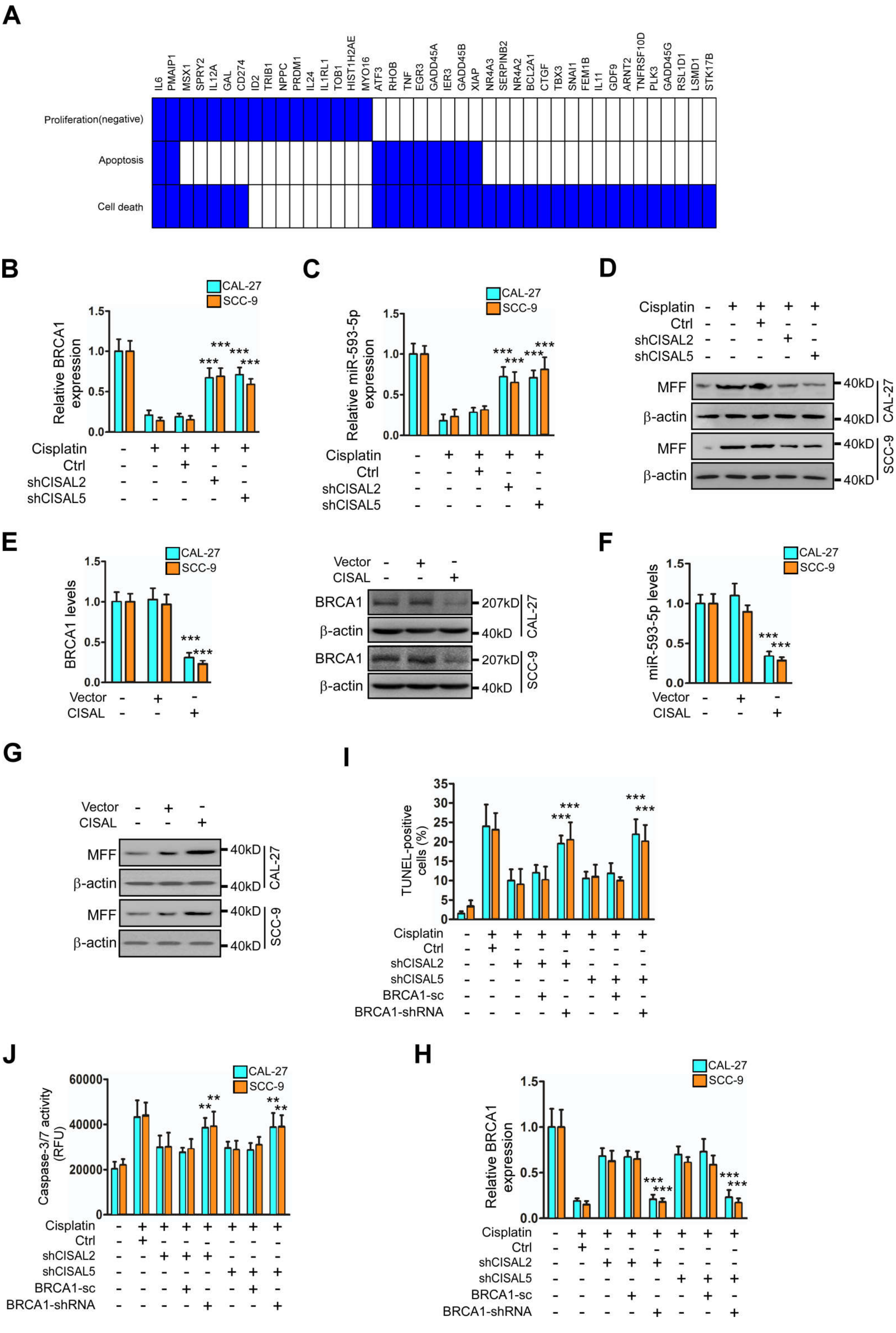

A
IL6
PMAIP1
MSX1
SPRY2
IL12A
GAL
CD274
ID2
TRIB1
NPPC
PRDM1
IL24
IL1RL1
TOB1
HIST1H2AE
MYO16
ATF3
RHOB
TNF
EGR3
GADD45A
IER3
GADD45B
XIAP
NR4A3
SERPINB2
NR4A2
BCL2A1
CTGF
TBX3
SNAI1
FEM1B
IL11
GDF9
ARNT2
TNFRSF10D
PLK3
GADD45G
RSL1D1
LSMD1
STK17B
Proliferation(negative)
Apoptosis
Cell death
B
CAL-27
SCC-9
Relative BRCA1 expression
Cisplatin
Ctrl
shCISAL2
shCISAL5
C
Relative miR-593-5p expression
D
MFF
β-actin
40kD
CAL-27
SCC-9
E
BRCA1 levels
Vector
CISAL
BRCA1
207kD
F
miR-593-5p levels
G
I
TUNEL-positive cells (%)
BRCA1-sc
BRCA1-shRNA
J
Caspase-3/7 activity (RFU)
H

Figure S5. CISAL regulates BRCA1, miR-593-5p and MFF expression in TSCC cells. (A) Gene set enrichment analysis (GSEA) showing three significantly induced pathways associated with the genes downregulated upon CISAL knockdown in both TSCC cell lines under cisplatin treatment. (B) qRT-PCR showing that knockdown of CISAL expression increases BRCA1 in TSCC cells under cisplatin treatment. (C) miR-593-5p was upregulated upon CISAL silencing in TSCC cells under cisplatin treatment. (D) Western blotting indicating that MFF expression was downregulated upon CISAL knockdown in TSCC cells under cisplatin treatment. (E) qRT-PCR (left panel) and Western blot (right panel) demonstrating that CISAL overexpression results in reduction of BRCA1 expression in CAL-27 and SCC-9 cells. (F) Overexpression of CISAL downregulated miR-593-5p expression in both TSCC cell lines. (G) The MFF protein level was increased by CISAL overexpression in both TSCC cell lines. (H-I) The inhibitory effect of CISAL knockdown on mitochondrial fission, analyzed by TUNEL (H) and caspase-3/7 activity (I), after BRCA1 silencing. (J) qRT-PCR showing that the enhanced expression of BRCA1 by CISAL knockdown was attenuated upon silencing of BRCA1. $^{**}P<0.01$ and $^{***}P<0.001$, 2-tailed Student's t test. Data are represented as mean ±SEM. Related to Figure 2.

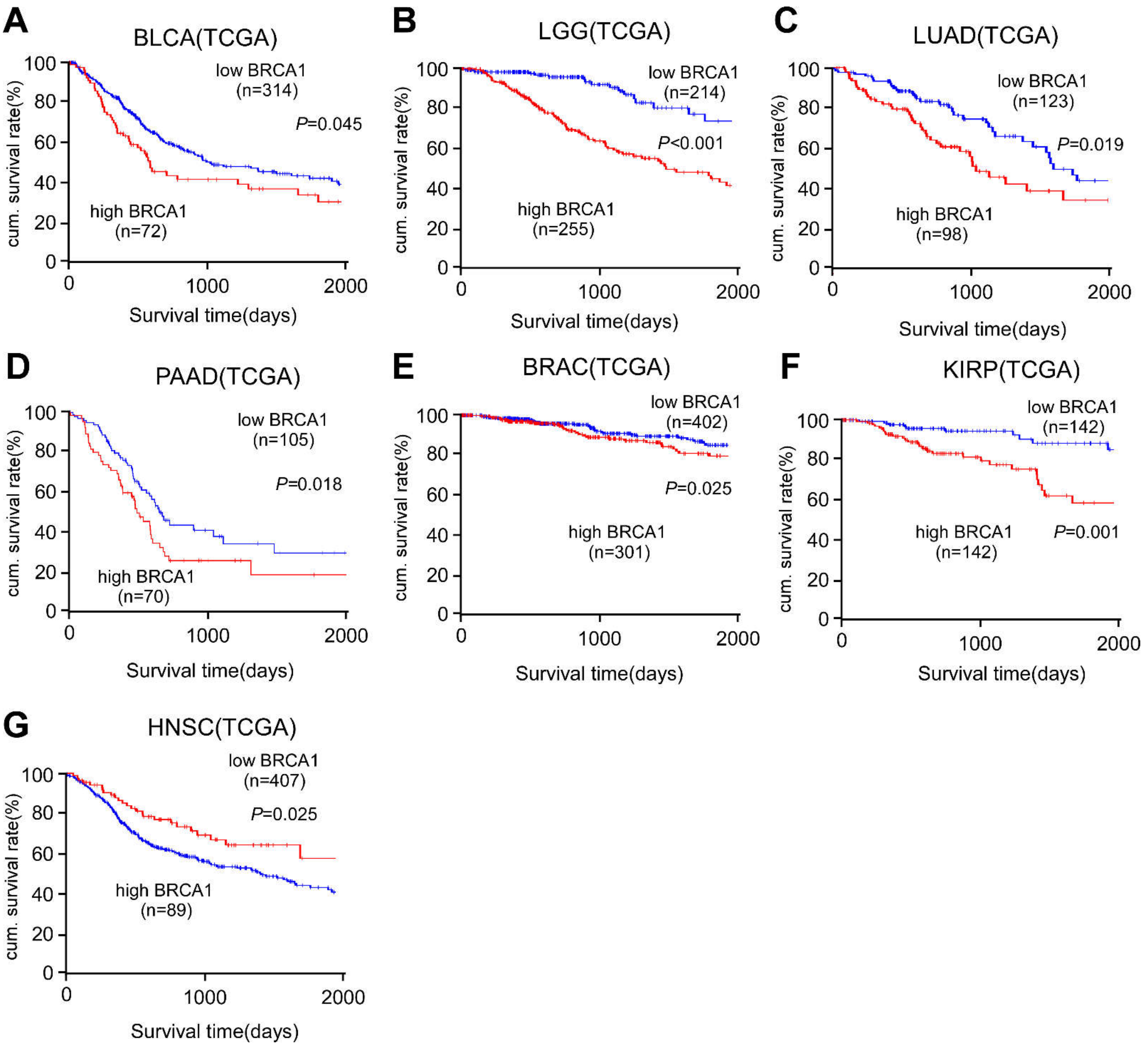

Figure S6. (A-G) TCGA data analysis showing that lower BRCA1 expression is associated with better overall survival in multiple types of cancer such as bladder carcinoma (BLCA) (A), low grade glioma (LGG) (B), lung adenocarcinoma (LUAD) (C), pancreatic adenocarcinoma (PAAD) (D), breast invasive carcinoma (BRAC) (E), kidney renal papillary cell carcinoma (KIRP) (F), head neck squamous cell carcinoma (HNSC) (G). Related to Figure 2.

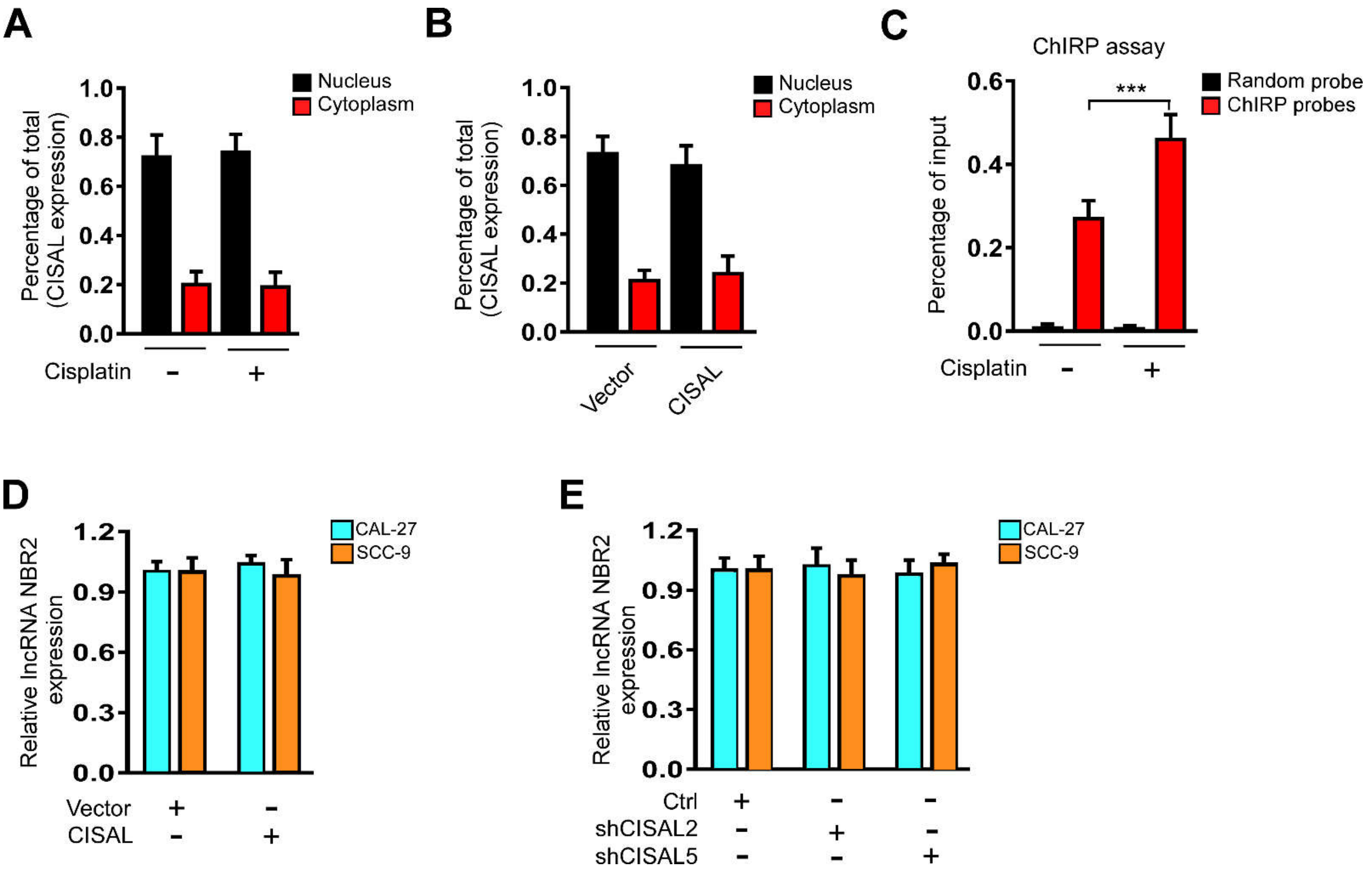

Figure S7. (A and B) CISAL distribution in cisplatin-treated CAL-27 cells (A) and untreated CAL-27 cells with stable CISAL expression (B). (C) ChIRP analysis of CISAL around the regulatory regions of BRCA1(-1627, -1606) in CAL-27 cells under cisplatin treatment. The crosslinked CAL-27 cell lysates were incubated with biotinylated DNA probes against CISAL, and the binding complexes were recovered using streptavidin-conjugated magnetic beads. qPCR was performed to detect enrichment of the specific regulatory regions associated with CISAL. (D and E) lncRNA NBR2 levels were detected by qRT-PCR in CAL-27 and SCC-9 cells with CISAL overexpression (D) or knockdown (E). ***P<0.001, 2-tailed Student's t test. Data are represented as mean ±SEM. Related to Figure 3.

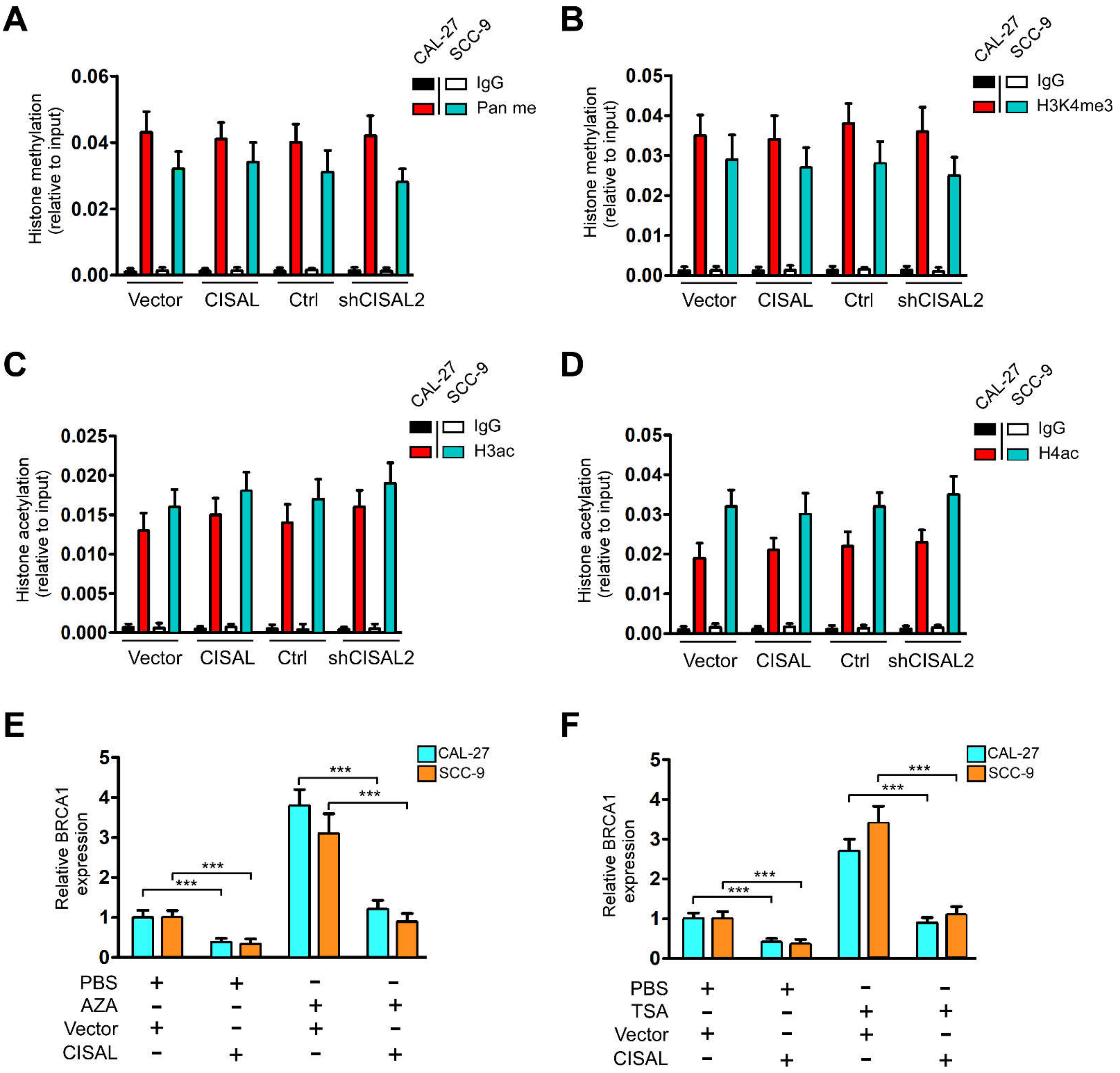

Figure S8. CISAL has no effect on histone methylation and deacetylation at BRCA1 promoter. (A and B) The pan-histone H3 methylation (Pan me) and H3K4 methylation (H3K4me3) at BRCA1 promoter regions were analyzed by ChIP-qPCR assay in CAL-27 and SCC-9 cells. (C and D) The histone H3 acetylation (H3ac) and histone H4 acetylation (H4ac) at BRCA1 promoter regions were analyzed by ChIP-qPCR assay in CAL-27 and SCC-9 cells. The histone modification in 10% input DNA was set to 1. IgG was used as a negative control. (E) CISAL overexpression reduced BRCA1 expression upon 5-aza-dC treatment. (F) CISAL overexpression reduced BRCA1 expression under TSA treatment. ***P<0.001, 2-tailed Student's t test. Data are represented as mean ±SEM. Related to Figure 4.

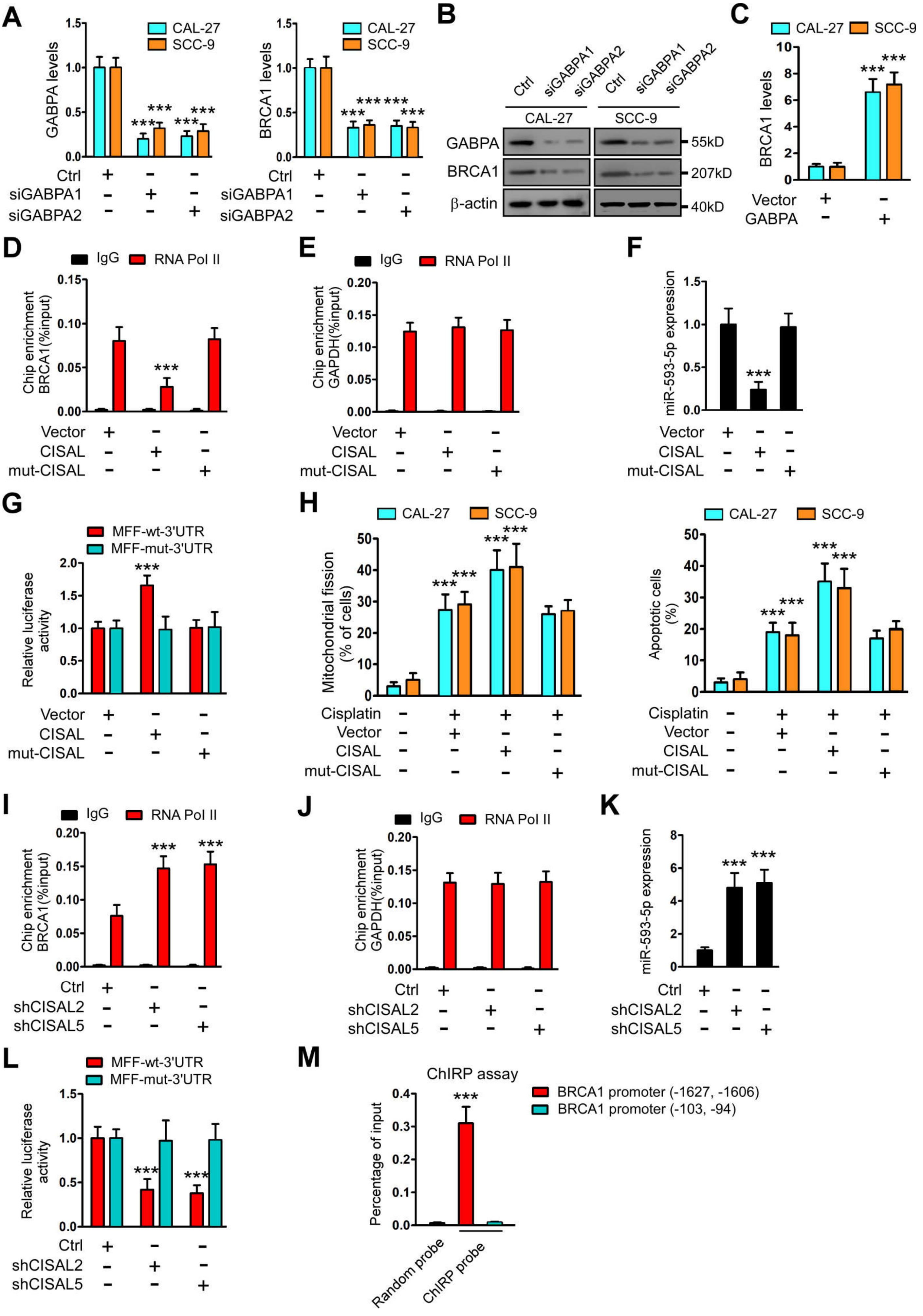

Figure S9. CISAL modulates BRCA1-miR-593-MFF axis through transcriptional regulation of BRCA

expression. (A and B) qRT-PCR (A) and Western blotting (B) showing that knockdown of GABPA significantly reduces GABPA and BRCA1 expression in CAL-27 and SCC-9 cells. (C) BRCA1 expression in CAL-27 and SCC-9 cells with stable GABPA expression. (D and E) ChIP-qPCR analysis of RNA pol II occupancy in the BRCA1 (D) and GAPDH (E) promoter after overexpression of CISAL or mutant CISAL (mut-CISAL) in CAL-27 cells. (F) Forced expression of mut-CISAL failed to inhibit miR-593-5p levels in CAL-27 cells. (G) Luciferase reporter assay demonstrating overexpression of CISAL but not its mutants reduces miR-593 functionality in CAL-27 cells. (H) Mutant CISAL failed to enhance mitochondrial fission and cell apoptosis in CAL-27 and SCC-9 cells. (I and J) ChIP-qPCR analysis of RNA pol II occupancy in the BRCA1 (I) and GAPDH (J) promoter in CAL-27 cells with CISAL knockdown. (K) Knockdown of CISAL expression increases miR-593-5p levels in CAL-27 cells. (L) Luciferase reporter assay showing that CISAL knockdown enhances miR-593 functionality in CAL-27 cells. (M) ChIRP analysis showing CISAL in the regulatory regions of BRCA1 promoter (-1627, -1606) but not (-103, -94). The crosslinked CAL-27 cell lysates were incubated with biotinylated DNA probes against CISAL, and the binding complexes were recovered using streptavidin-conjugated magnet beads. qPCR was performed to detect enrichment of the specific regulatory regions associated with CISAL. ***P<0.001, 2-tailed Student's t test. Data are represented as mean ±SEM. Related to Figure 4.

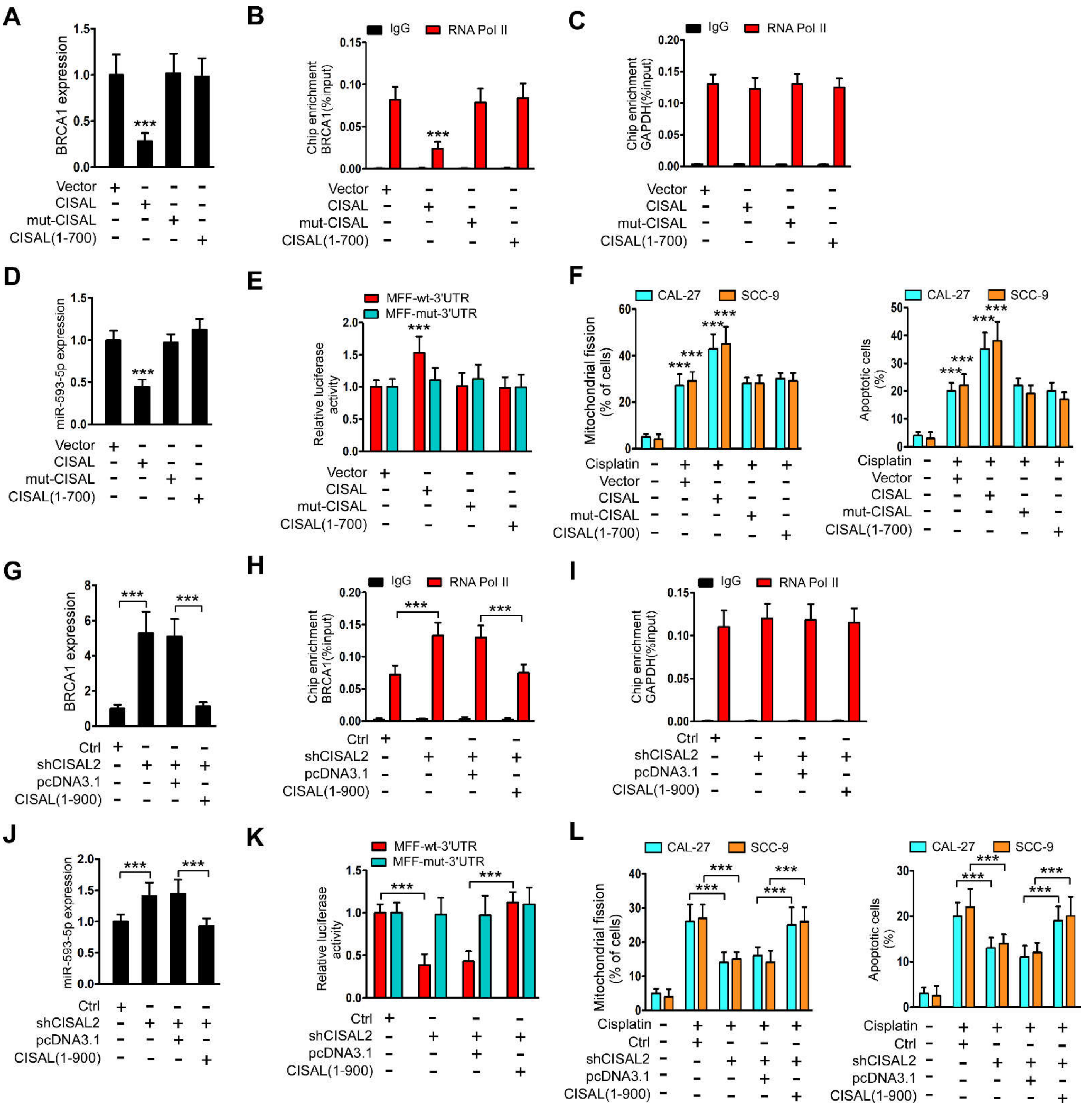

Figure S10. CISAL determines BRCA1 transcription, mitochondrial fission and cell apoptosis in TSCC cells. (A) Site-directed mutagenesis of 1–700 nt of CISAL leads to a loss of the effect on BRCA1 levels in CAL-27 cells. (B and C) ChIP-qPCR analysis demonstrating that forced expression of truncated CISAL (1–700) had no effect on RNA pol II occupancy in the BRCA1 (B) and GAPDH (C) promoter in CAL-27 cells. (D and E) Overexpression of CISAL (1–700) lost ability to inhibit miR-593-5p expression (D) and the functionality (E) in CAL-27 cells. (F) Mitochondrial fission and apoptosis were detected in both CAL-27 and SCC-9 cells with transduction of site-directed mutagenesis of 1–700 nt of CISAL. (G) Forced expression of the truncated CISAL (1–900) abolished the increase of BRCA1 levels in CAL-27 cells. (H and I) ChIP-qPCR analysis showing that forced expression of the truncated CISAL(1–900) abolishes the increase of RNA pol II occupancy in the BRCA1 (H) but not GAPDH (I) promoter by depletion of endogenous CISAL in CAL-27 cells. (J and K) qRT-PCR (J) and luciferase assays (K) demonstrating that

forced expression of CISAL(1–900) attenuates the increase of miR-593-5p levels and functionality by depletion of endogenous CISAL in CAL-27 cells. (L) Forced expression of the truncated CISAL (1–900) abolished the decrease of mitochondrial fission and apoptosis in both CAL-27 and SCC-9 cells by silencing endogenous CISAL. ****P*<0.001, 2-tailed Student's t test. Data are represented as mean ±SEM. Related to Figure 5.

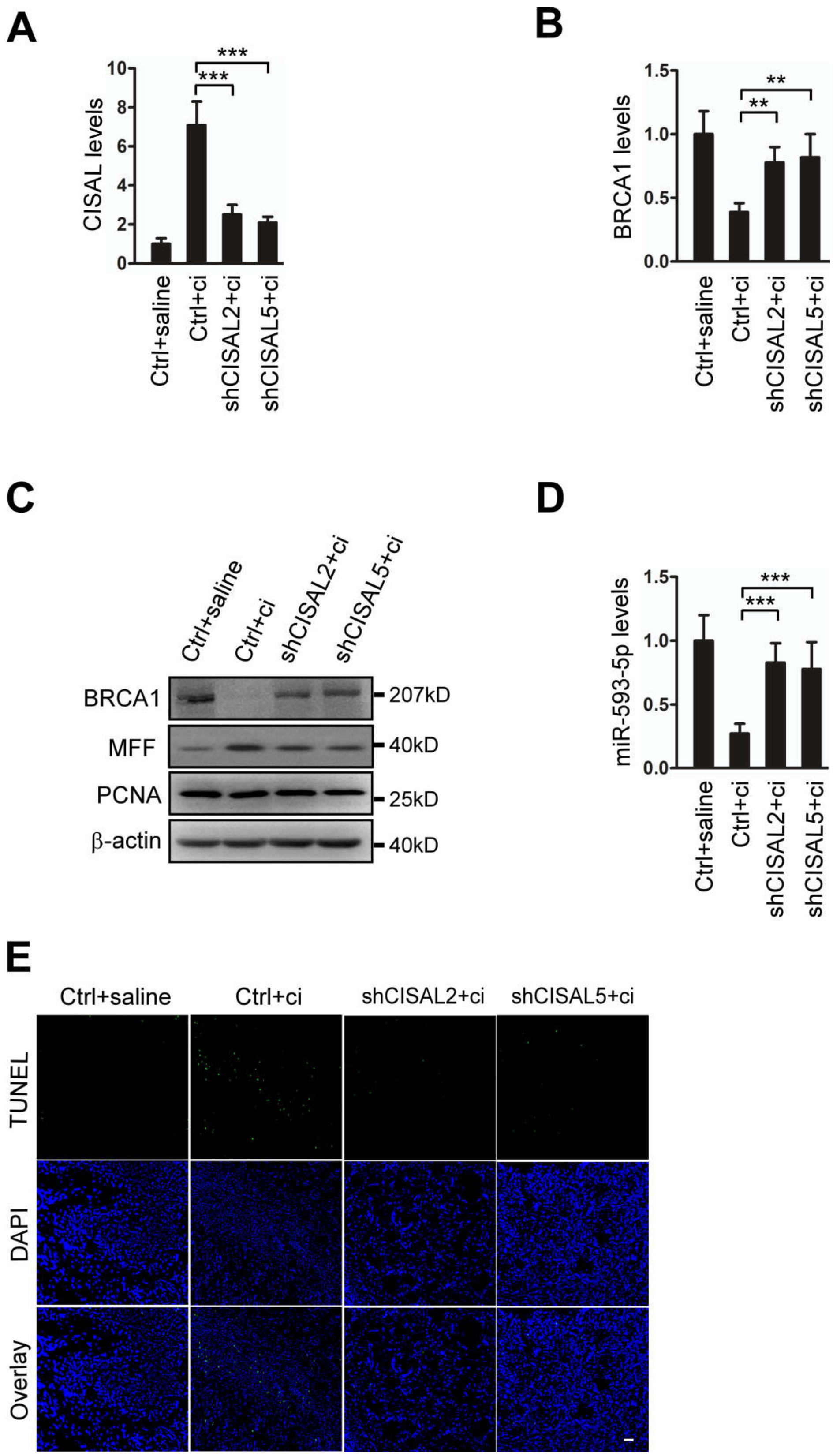

Figure S11. CAL-27 xenograft with CISAL knockdown in BALB/c nude mice. (A) qRT-PCR showing CISAL expression in each group. (B) qRT-PCR showing that BRCA1 expression was increased by CISAL knockdown. (C) Western blot indicating that BRCA1 expression was increased, but MFF was downregulated, upon silencing CISAL in TSCC xenograft treated with cisplatin, while PCNA was not changed in any group. (D) qRT-PCR showing that miR-593-5p expression was upregulated upon CISAL knockdown. (E) TUNEL assays showed that apoptosis in response to cisplatin was attenuated by CISAL knockdown. scale bar, 20 μm.***P*<0.01 and ****P*<0.001, 1-way ANOVA followed by Dunnett's tests for

multiple comparisons. Data are represented as mean ±SEM. Related to Figure 6.

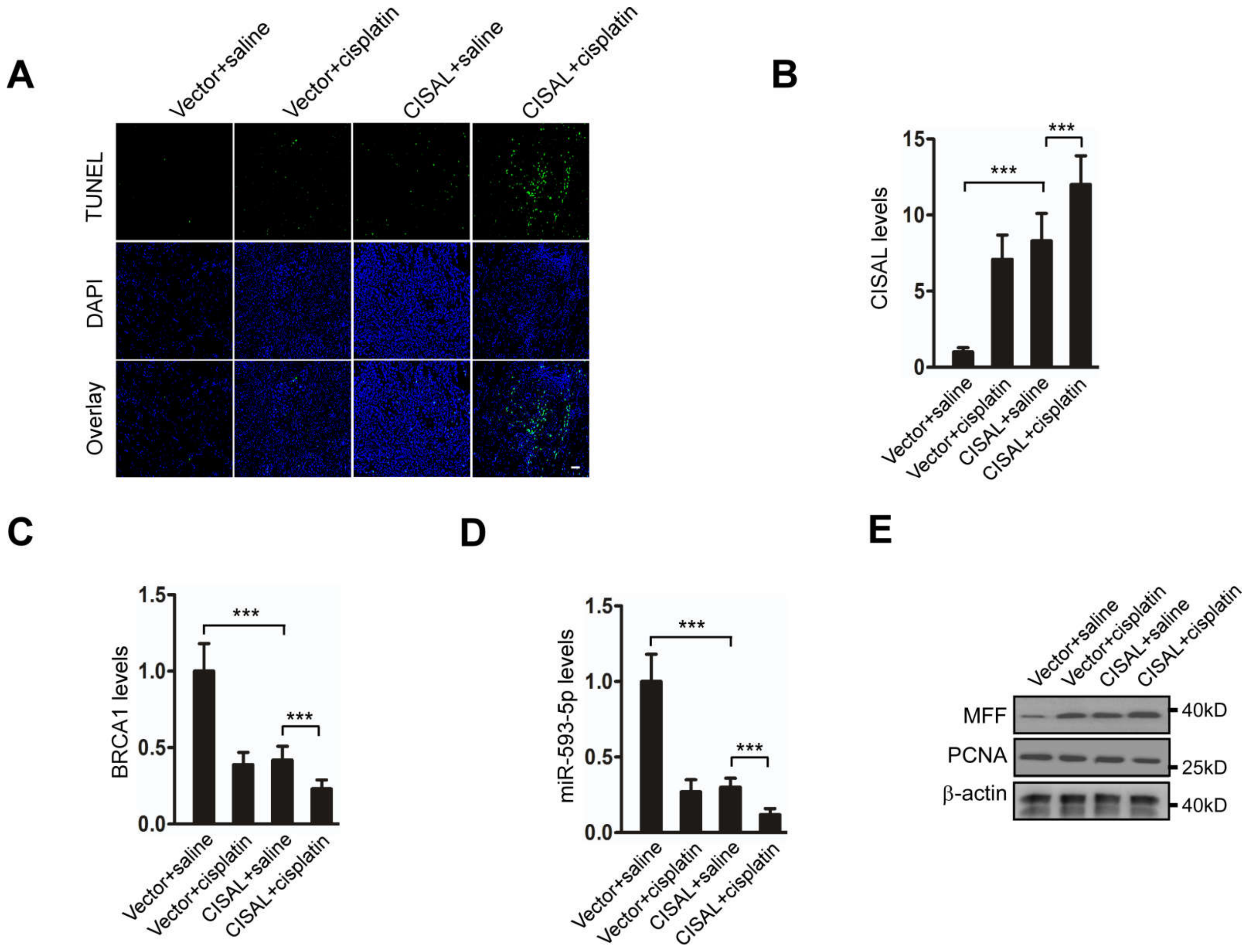

Figure S12. CISAL overexpression increases apoptosis and cisplatin sensitivity in CAL-27 xenografts in vivo. (A) BALB/c nude mice bearing xenografts of CAL-27 cells with stable CISAL expression or control vector were treated with saline or cisplatin (n=6 per group). TUNEL assay showing that apoptosis in response to cisplatin was attenuated upon CISAL knockdown; scale bar, 20 µm. (B-E) CISAL (B), BRCA1 (C), miR-593-5p (D), MFF and PCNA (E) expression in each group. ****P*<0.001, 1-way ANOVA followed by Dunnett's tests for multiple comparisons. Data are represented as mean ±SEM. Related to Figure 6.

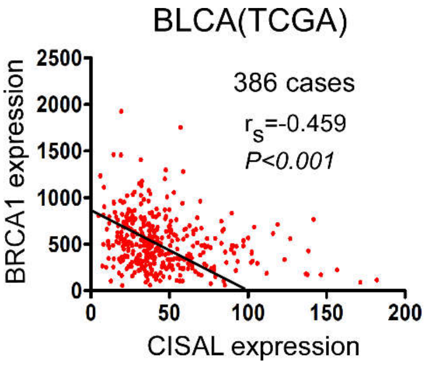

Figure S13. TCGA data analysis showing that CISAL levels reversibly correlates with BRCA1 expression in bladder carcinoma (BLCA). Related to Figure 7.

Supplemental Tables

Table S1. Clinical characteristics of TSCC patients. Related to Figure 1.

Patient ID	Age (years)	Gender	Pathological diagnosis	TNM stage	NaC	Cycles	Responses
PS 1	69	F	TSCC	T4N1M0	TPF/TPF	2	Partial
PS 2	56	F	TSCC	T3N0M0	TPF	1	Partial
PS 3	43	F	TSCC	T4N1M0	TPF/TPF	2	Partial
PS 4	73	M	TSCC	T3N1M0	TPF	1	Partial
PS 5	66	F	TSCC	T3N0M0	TPF/PF	2	Partial
PS 6	54	F	TSCC	T3N1M0	TPF/TPF	2	Partial
PS 7	41	M	TSCC	T3N0M0	TPF/TP	2	Partial
PS 8	77	M	TSCC	T4N2M0	TPF	1	Partial
PS 9	59	F	TSCC	T3N0M0	TPF/TPF	2	Partial
PS 10	68	M	TSCC	T3N1M0	TPF	1	Partial
PS 11	71	F	TSCC	T4N1M0	TPF/TP	2	Partial
PS 12	54	M	TSCC	T4N2M0	TPF	1	Partial
PS 13	39	M	TSCC	T3N1M0	TPF/TP	2	Partial
PS 14	48	F	TSCC	T4N2M0	TPF/TP	2	Partial
PNS 1	54	M	TSCC	T3N0M0	TPF/TPF	2	Progressive
PNS 2	67	F	TSCC	T3N2M0	TPF	1	Stable
PNS 3	48	M	TSCC	T3N1M0	TPF/TPF	2	Progressive
PNS 4	49	F	TSCC	T4N2M0	TPF	1	Progressive
PNS 5	35	F	TSCC	T3N0M0	TPF/TPF	2	Stable
PNS 6	55	M	TSCC	T4N1M0	TPF	1	Stable
PNS 7	39	F	TSCC	T3N0M0	TPF	1	Progressive
PNS 8	41	F	TSCC	T3N1M0	TPF/PF	2	Stable
PNS 9	67	M	TSCC	T3N1M0	TPF/TPF	2	Stable
PNS 10	59	M	TSCC	T3N0M0	TPF/TPF	2	Progressive
PNS 11	68	M	TSCC	T4N2M0	TPF	1	Stable
PNS 12	69	F	TSCC	T3N0M0	TPF/TPF	2	Progressive
PNS 13	74	M	TSCC	T3N0M0	TPF/TPF	2	Stable
PNS 14	53	M	TSCC	T4N0M0	TPF	1	Stable
PNS 15	47	F	TSCC	T4N2M0	TPF/PF	2	Stable

PS, patient with partial response; PNS, patient with progressive or stable disease; TSCC, tongue squamous cell carcinoma; NaC, neoadjuvant chemotherapy; TP, docetaxel+cisplatin; PF, cisplatin+ 5-fluorouracil; TPF, docetaxel+cisplatin+5-fluorouracil;

Table S2. The full-length nucleotide sequence of CISAL. Related to Figure 1.

GACTTGGACCAGCAGGGGAATGGCTGTGACAATAAATAAGATTGGGAAAACAAGAAGACGCCC
TTGCATACATTAAGATATGTATATATGGAAATGTTACCGGGAGTCCCGGTTATCCCAAAAAGGGTT
GTTTCCTGTTGCGTGGTGAGGCCAATGCACGAAACCGAAAGGGAGTGTGTCAAGCAGTGCAGG
CTGTATTCAATGGCTATGGAATTGGAAGATCTGAAATCAACTTAGCTTGTGAGAGCTGGGAAGTT
TCAGAGTCGTTGTCATGGCGACATGTTGTTTAGCATGAGAACAGGATGATGATAAAGCCAGAGG
CTCTTCAGAGGCGCCATCTTGGATTTCGCCAGCTTCAGCTGGTTTCGTCCTAAGAAGGAACTTCG
AAACACAGGCATTCTTTTTCCTGAAAATAAGCAGAGTTACAGCTGAGTAGGAATTTAGCTCTGTC
CCATATGCTATCGCATTGGGCAGCAAAAGCAGGGTGGGGTCCAGCGAAATCAGCAGGCACTGCA
ATGAGTAACATACCCAGCCACGTTTATGCAGCATTTTTACGAAAATGAAACCATACTACCTGTAA
AGGAAGATATGCTAACAAACAACAAAACTGGCAGGAACCAAGATTCCTACTGACACTACCCTTA
GTTTTAATTTTCCCTGACAACAAATGAGGTTAACAGAGCATAATTATCTACCGTGACCCCTTCAA
AAAGACAGGCTGTATACATTTGCACTAAGAGAAGAAATCGTGTAATGTCAGCAAATTTCCCCCA
CTTAAAGCTTCTCTATTTAAAAAGCTTCACGCACACATGCACGCATATGTCTTCAAGATGACCAC
AATTTATTTTGCAGTCATTCTTTGCACCAGTTCCCAATTTTTCCCACTAGCTTGCAAGCTCCGTGA
CTCGAGGAGACCGGGGGATCAGAGTTTGTTTTTGCGGAGAAGTGAGTCCTTTATGCCCCAAATA
GTGACTGACATAGAGAAGGTACTCAGTAAACACTTTTTAAAGGAATGCCTGCCTGACTGAAGCT
TAATGATGTGAGGCTTCTAGTGGGATACCCTACCTTGTTTTAACCTGAAGTGACTCTTCCTTAGCT
AAGAGAGCCAGACGGACTCCATCGTGACTCCTTCACTCGCAGCCCCTTACCCACCCCCTTCCTC
AAGGACTTAACTTGTGCAAGCTGACTCCCAGCACATCAAGAATGCAATTAACTGATAAGATACT
GTGGCAAGCTATATCCGCAGTTCCCAGGAATTCGCCCGGTTAATAGCACCCAGAGCCCCTGCGTT
TGTGTCCGGTTGATAACGCCCAAAGCCCGGCGTCCATCACCTTAGGATAGACTTAAAGCCTCTGC
ACCTGGAACTGTTTACTTTCCTGTAACCGTTTATCCTTTTAACTTTTTGCCTACTTTACTTCTGTAA
GATTGTTTCAACTAGACTCCCCCTCTCCCCTGTCTAAACCAAAGTATAAAAGAAAATCTAGCTCC
TTCTTCGGGGCCAAGAGAATTTCGAGCGCTAGCTGTCTCTCGGCTGCCGGCTAATAAAGGACTC
CTGAATTCGTCTCAGAAAAAAAAAAAAAAAA

Table S3. Correlation among clinicopathological status and the expression of CISAL and BRCA1 in TSCC patients. Related to Figure 7.

Characteristics	CISAL (%)		*P*	BRCA1(%)		*P*
	No. of low Expression	No. of high Expression		No. of low Expression	No. of high Expression	
**Sex**			0.770			0.234
Male	26(41.9)	36(58.1)		42(67.7)	20(32.3)	
Female	20(39.2)	31(60.8)		29(56.9)	22(43.1)	
**Age**			0.980			0.120
＜50	15(40.5)	22(59.5)		27(73.0)	10(27.0)	
≥50	31(40.8)	45(59.2)		44(57.9)	32(42.1)	
**Node metastasis**			0.959			0.329
N0	19(40.4)	28(59.6)		32(68.1)	15(31.9)	
N＋	27(40.9)	39(59.1)		39(59.1)	27(40.9)	
**Clinical stage**			0.845			0.232
Ⅲ	28(40.0)	42(60.0)		41(58.6)	29(41.4)	
Ⅳ	18(41.9)	25(58.1)		30(69.8)	13(30.2)	
**Cisplatin**			0.009			＜0.001
Sensitive	18(29.5)	43(70.5)		48(78.7)	13(21.3)	
Non-sensitive	28(53.8)	24(46.2)		23(44.2)	29(55.8)	
**Status (60 months)**			0.018			0.008
Survival	10(25.6)	29(74.4)		31(79.5)	8(20.5)	
Death	36(48.6)	38(51.4)		40(54.1)	34(45.9)	

Table S4. Primers used in this study. Related to Figure 1.

Gene	Forward primer (5'-3')	Reverse primer (5'-3')
**qRT-PCR**		
NR_033928	TTCTTTCACCATTTCACACA	AACCCTACCTGGACACATC
TCONS_00019861	CAGAGAATGCGTGAATGG	TTGAGGGAAGGGGAGTAA
TCONS_00014652	GCTTTCTTTCACCATTTCAC	GGACACATCATAGTCATCATACA
ENST00000558994	GACGGGAATGAAAAGAAGA	CAGAGAGGTGGAGGGACT
CISAL	CGAAACCGAAAGGGAGTG	CATGTCGCCATGACAACG
NR_034085	GCAGTATGAAAATGGCTAGAGATTG	GGCTCTACATCATTTGTCGTATGTG
ENST00000449527	ACTTTCTTCAGTCACATCTGAA	CGCCAATGGGTGAAATCTAAAG
uc003hxs.3	AGGCGTAAGAAACCTCCT	TTCATCAGAGTCCCTCCA
NR_001458	GAACAACCTACCAGAGACCTT	CACAGATTTCCCCTTCCT
uc001dqd.1	TACACCCTTGAATCCCCTCTT	TCCATTGTTCCCTAACGACC
uc010kun.2	TCATCCATTCTTCACCGA	CATTTTCATTTTCACACCAA
ENST00000450469	GCCTGTGGGTAGATTTGA	TGTATTTTGATGACCTCTGCT
ENST00000581816	AACTCAAACCCCTTTCTACAC	ATCCCCACCAAACTCAAC
ENST00000559960	GTTCCAGCATAAAGTGAAAGA	AAGACAGCAAAACCAAAGAG
TCONS_00010989	GAAAGGAAGAAGGGAGAGAA	AGGATTACAGGCGTGAGC
TCONS_00027385	TCTATTTTGTCTCACCAGCAC	GCCTCAGTTTCCCTCACT
ENST00000578792	AAAATCAGGACGGAAAGG	CGACCCAGACTATTGGAG
ENST00000440578	ACGAGGTGGGAAGAAAAC	GAGAACAAAACAGTGAACAGAG
ENST00000440397	GCTTGTTTGGTTTCTGATAGTT	CCTCCTCTCCCTGGTATG
ENST00000464115	CGAGATGGTGGTGAATGT	AGTCCGAAGCGAGAGAAG
ENST00000577281	CACCCTCATCCCACTCTC	CCCACCATCTCACTTTCA
NR_037597	ATTTGTGTGTTGGATGGTG	CTTGATTTTGGACTTGTGG
uc010shg.2	CAAAAGATAAAGATGAACAGGAA	AAAGGAAGGAACAACCACTC
ENST00000424612	AGGAAACAAAAGCAAAACTG	GCCAGGAATAAAGCGAAG
ENST00000430320	AACTTTTATCAGCGGCAGT	GTTGGCTTCCATCTTGTG
TCONS_00011960	AGCACTGGACACACAAGAG	ATCACAGACCACAGCAGAA
ENST00000444326	TCATAGAGCCCAGAACAAA	GAAGCACAACCAGATAGAAAA
ENST00000545308	CTGCCTCCCAGGGTGAAT	ATGGATGAGGGTAACAGCACA
ENST00000579480	AGGGAGGTTGCTGATTCT	AGTTGGTTATGGGCGTGT
ENST00000460249	CCTGTCCTTACTCCCTCTTT	GGCTTACCTTCTCTTGGG
ENST00000544983	CCGTGAGGATAAATAACTCTG	GACAGGAGCCCAATAAGAC
uc001nvs.3	CCAGAACCCAAACTCAGG	ACAGAGGAACAGACACGAAG

ENST00000427501	GTTCTGAGTGTGGACGAGTAG	AGGTAATGCTAAAAGGCAAGT
ENST00000535076	CCAGAACCCAAACTCAGG	ACAGAGGAACAGACACGAAG
ENST00000532315	CCTCTGTCTGTCCTGCCT	CAGTCTCTTCAGTCTTTGTCCT
ENST00000431043	GTTCTGAGTGTGGACGAGTAG	AGGTAATGCTAAAAGGCAAGT
uc009zhn.3	CAAAAGATAAAGATGAACAGGAA	AAAGGAAGGAACAACCACTC
ENST00000506274	TTAGCGACATCAGGAAGAAC	CAAAGGAAGAGGGGACTG
BRCA1	GGCTATCCTCTCAGAGTGACATTTTA	GCTTTATCAGGTTATGTTGCATGGT
lncRNA NBR2	GGAGGTCTCCAGTTTCGGTA	TTGATGTGTGCTTCCTGGG
U6	CTCGCTTCGGCAGCACA	AACGCTTCACGAATTTGCGT
β-actin	AGCCTCGCCTTTGCCGATCC	ACATGCCGGAGCCGTTGTCG
**Plasmid construction**		
pcDNA-GABPA	ATAGGTACCATGACTAAAAGAGAAGCAGAGGAG	ATTGCGGCCGCTCAATTATCCTTTTCCGTTTGC
pcDNA-CISAL-wt	AAGGATCCAGACTACTGACTTGGACCAGCA	TCGATATCCTGAGACGAATTCAGGAGTCCT
pcDNA-CISAL-mut	ATCGTGACTCCTTCAAGACAUCAGAGGAUGAGUGCCCCTTCCTCAAGGAC	GTCCTTGAGGAAGGGGCACTCATCCTCTGATGTCTTGAAGGAGTCACGAT
pGL3-BRCA1	TGTGGTACCTGCATTTGCAAACCTTGAGC	TACTCGAGAGAGGGTGAAGGCCTCCTGA
**EMSA**		
BRCA1-70	GGGAGGCTCAGGCCACGCT	AGGACCTGCAGCCCGCCA
BRCA1-120	GCGCTGAGGAGCAGGGG	CCCTGCACAGGGCAAGGCT
**ChIP-qPCR**		
BRCA1(Histone Me/Ac)	AGGGCAGGCACTTTATGGC	CGCAGTCGCAGTTTTAATTTATC
BRCA1-GABPA	AGGGCAGGCACTTTATGGC	TACGAAATCAAGGTACAATCAGAGG
GAPDH	GACCTTCTTGCCTTGCTCTTG	GCCTGCCTGGTGATAATCTTT
**ChIRP**		
BRCA1-CISAL (-1627,-1606)	CTAGACATAAAAGTTTTCCAAGTCCC	ACAGGGCAAGGCTCAGGACC
BRCA1-CISAL (-103,-94)	AGGGCAGGCACTTTATGGC	TACGAAATCAAGGTACAATCAGAGG

BRCA1(distal)	TTTGTTCGTTCCTCCCGTCT	CTCTGGTCTCCTTCCACGCT
GAPDH(proximal)	GACCTTCTTGCCTTGCTCTTG	GCCTGCCTGGTGATAATCTTT
GAPDH(distal)	TCCCCAATTTCATTCCCTTTA	CGCAGATGCCACGGATTAGTT
**5' RACE**	**Sequence (5'-3')**	
UPM (10×) Primer	CTAATACGACTCACTATAGGGCAAGCAGTGGTATCAACGCAGAGT	
RACE assay-outer	CCACCAGCGCCGTGACAACTGAC	
UPS (10 μM) Primer	CTAATACGACTCACTATAGGGC	
RACE assay-inner	CAGCCTGCACTGCTTGACACACTC	
**3' RACE**	**Sequence (5'-3')**	
3' Outer Primer	TACCGTCGTTCCACTAGTGATTT	
RACE assay-outer	AAGTGACTCTTCCTTAGCTAAGAG	
3' Inner Primer	CGCGGATCCTCCACTAGTGATTTCACTATAGG	
RACE assay-inner	ATCCGCAGTTCCCAGGAATTCGC	

Table S5. Sequences of shRNAs/siRNAs and probes used in this study. Related to Figure 3

	**Sequence(5'-3')**
**CISAL shRNA**	
shRNA #1	CCAATGCACGAAACCGAAA
shRNA #2	TGGCTATGGAATTGGAAGA
shRNA #3	TTGCACTAAGAGAAGAAAT
shRNA #4	CTCCTGAATTCGTCTCAAA
shRNA #5	CCTACTGACACTACCCTTA
shControl	TCTTAATCGCGTATAAGGC
**GABPA siRNA**	
siGABPA1	GGAGCTGATAGAAATTGAGATTGAT
siGABPA2	GCAGAGTGCACAGAAGAAAGCATTG
**RNA oligonucleotides**	
Oligo #1	AGAGCCAGACGGACUCCAUC
Oligo #2	ACUCCAUCGUGACUCCUUCA
Oligo #3	CUCCUUCACUCGCAGCCCCU
Oligo #4	CAGCCCCUUACCCACCCCCU
Oligo #5	CACCCCCUUCCUCAAGGACU
Oligo-wt	GGACUCCAUCGUGACUCCUUCACUCGCAGCCCCU UACCCACCCCCUUCCUCAAGGACUUAACUUGUGC
Oligo-mut	GGACUCCAUCGUGACUCCUUCAAGACAUCAGAGG AUGAGUGCCCCUUCCUCAAGGACUUAACUUGUGC
**ChIRP probes**	
ChIRP #1	GTGTCAGTAGGAATCTTGGT
ChIRP #2	GAAGCCTCACATCATTAAGC
ChIRP #3	AGTCTATCCTAAGGTGATGG
ChIRP #4	GAACTGGTGCAAAGAATGAC
ChIRP #5	AAAGAATGCCTGTGTTTCGA
ChIRP #6	TCATCCTGTTCTCATGCTAA
ChIRP #7	TGCTGACATTACACGATTTC
ChIRP #8	GGCATAAAGGACTCACTTCT

ChIRP #9	GGCTGGGTATGTTACTCATT
Random probe	TGGGAGTGTTTATACGCGTA
**In situ hybridization (ISH)**	
CISAL	TAAGCTTCAGTCAGGCAGGCAT
Scramble	GTGTAACACGTCTATACGCCCA
U6	CACGAATTTGCGTGTCATCCTT
β-actin	CTCATTGTAGAAGGTGGGTGCCA

Transparent Methods

Cell culture

Two human TSCC cell lines, CAL-27 and SCC-9, were obtained from the American Type Culture Collection. CAL-27 cells were cultured in Dulbecco's modified Eagle's medium (Gibco, Rockville, MD, USA) supplemented with 10% fetal bovine serum (Invitrogen, Carlsbad, CA, USA). SCC-9 cells were cultured in Dulbecco's modified Eagle's medium-F12 (Gibco) supplemented with 10% fetal bovine serum. Cells were cultured at 37°C in a humidified atmosphere containing 5% CO2. For drug treatment, cisplatin (Sigma, USA), adriamycin (ADR) (Sigma, USA) or camptothecin (CPT) (Sigma, USA) were administered at a dose corresponding to their IC50(Fan et al., 2015a; Fan et al., 2015b) for 2 h, while 5-aza-2′-deoxycytidine (5-aza-dC) (Sigma, A3656) and trichostatin A (TSA) (Sigma, T8552) were administered for 72 h at 100 ng/mL(Wei et al., 2005) and the medium was changed every 24 h.

Electrophoretic mobility shift assay (EMSA)

EMSA was performed as previously described with slight modifications(Schmitz et al., 2010). Briefly, a total of 0.5 pmoles of a biotin-labeled DNA fragment (BRCA1-70, from -1655 to -1586; BRCA1-120, from -1685 to -1566) was obtained by PCR and incubated with 10 pmoles of synthetic RNA oligonucleotides (Table S 5) in 10 mM Tris-HCl (pH 7.4), 25 mM NaCl, 10 mM $MgCl_2$, and 10% glycerol for 2 h at room temperature. Then, 30 U of RNase H (Invitrogen™, 18021071) or RNase A (Thermo Scientific™, R1253) was used to test the resistance of DNA:RNA triplexes to RNase digestion. Triplex formation was monitored by electrophoresis on 8% polyacrylamide gels, followed by transfer to a nylon membrane and development using the BrightStar® BioDetect™ Kit System (Ambion).

Chromatin immunoprecipitation (ChIP)

ChIP assays were performed as previously described(Fan et al., 2015b). Briefly, CAL-27 cells (5×10^6) were washed with PBS and incubated for 10 min with 1% formaldehyde at room temperature. Crosslinking was halted with 0.1 M glycine for 5 min. The cells were washed twice with PBS and lysed for 1 h at 4°C in a lysis buffer, then sonicated into chromatin fragments with an average length of 500-800 bp, as accessed via agarose gel electrophoresis. The samples were precleared with Protein-A agarose (Roche) for 1 h at 4°C on a rocking platform. Then, 5 μg of specific antibodies was added and the samples rocked overnight at 4°C. Immunoprecipitated DNA was purified using the QIAquick PCR purification kit (Qiagen) according to the

manufacturer's protocol. The final ChIP DNA was then used as a template in qPCR with the primers in Table S 4. ChIP-grade anti-RNA polymerase II antibody (Abcam, ab5131), anti-GABPA antibody (Millipore, ABE1047), anti-pan methylated lysine antibody (Abcam, ab7315), anti-histone H3 (tri methyl K4) antibody (Abcam, ab8580), anti-acetylated histone H3 (Abcam, ab47915), anti-acetylated histone H4 (Millipore, 06–866), anti-GAL4(DNA binding domain) antibody (Millipore, 06-262), and anti-IgG (Sigma, I5006) were used in this study.

Chromatin isolation by RNA purification (ChIRP)

ChIRP assays were performed as previously described with slight modifications(Chu et al., 2012). Briefly, ChIRP probes (3'-end biotin labeled) against CISAL and the random probe were designed and synthesized by RiboBio (Guangzhou, China). CAL-27 cells were fixed with 1% glutaraldehyde for 10 min. The crosslinked cells were lysed with lysis buffer (50 mM Tris-Cl, pH 7.5, 10 mM EDTA, 1% SDS protease inhibitors and SUPERase-In). The lysates were sonicated by Bioruptor (Diagenode, Denville, USA) at 4 ℃ on the setting with pulse intervals of 30 seconds ON and 45 seconds OFF for a total of 30 min. The sonicated cell lysates were hybridized with a mixture of biotinylated DNA probes against human CISAL in hybridization buffer (50 mM Tris-Cl pH 7.5, 750 mM NaCl, 1% SDS, 1 mM EDTA, 15% formamide, protease inhibitors and SUPERase-In) overnight at 4℃. Then, the binding complexes were recovered by streptavidin-conjugated C1 magnetic beads (Invitrogen, Waltham, USA), and DNA was eluted with elution buffer (50 mM $NaHCO_3$, 1% SDS). Quantitative PCR was performed to detect the enrichment of specific regulatory regions associated with CISAL, and the percentage enrichment of the locus over the input DNA was determined.

RNA pull down assay

Full-length CISAL and antisense CISAL sequences were prepared by in vitro transcription using TranscriptAid T7 High Yield Transcription Kit(Thermo Scientific, K0441) and treated with RNase-free DNase I and purified with GeneJET RNA purification kit(Thermo Scientific, K0731). Nuclear extracts were prepared with NE-PER Nuclear Protein Extraction Kit (Thermo Scientific, 78833). For *in vitro* translated protein, full length of ORF fragment of a specific gene was inserted into pcDNA3.1 and was *in vitro* transcribed and translated with 1-Step Human Coupled *In Vitro* Expression Kit (Thermo Scientific, 88881) following standard protocols. RNA

pull down assay was performed with Magnetic RNA-Protein Pull-Down Kit (Pierce, 20164) according to manufacturer's instructions. Three micrograms of biotin-labeled RNA and 1 mg of nuclear extract were used in each pull down assay. The retrieved protein was detected with standard immunoblot technique.

RNA immunoprecipitation (RIP)

RIP was performed using a Magna RIP RNA-Binding Protein Immunoprecipitation Kit (Millipore, 17-700) according to the manufacturer's instructions. Briefly, $1x10^7$ cells were harvested and lysed with RIP lysis buffer for 20 min at 4°C. When the cell extract was removed from the dish, it was centrifuged for 15 min at 12000 g and 4°C. The supernatant was divided into two parts, and then, anti-GABPA antibody (Millipore, ABE1047) and IgG were added, followed by rotation at 20 rpm for 1 h. Protein beads were added to each tube followed by rotation at 20 rpm for 0.5 h. A magnetic frame was applied to remove the supernatant, followed by three washes with a lysis buffer. Protease K and RNase inhibitor were added to the lysis buffer, followed by rotation for 30 min at 55°C to remove the protein. TRIzol LS was added to the remaining solution, and RNA was then extracted from it. qRT-PCR was applied to assess CISAL expression in the immunocomplex.

***BoxB*-λN tethering assay**

As previously described(Wang et al., 2011b), the *BoxB* tethering system uses viral RNA–protein interactions, in which *BoxB* (GGGCCCUGAAGAAGGGCCC) is a viral RNA that can be recognized and bound by the viral anti-terminator protein λN (1-22: MDAQTRRRERRAEKQAKWKAAN). Fusion of CISAL with *BoxB* enables the fused CISAL-*BoxB* to be bound by λN. Subsequently, λN protein is fused with the DNA-binding domain (DBD) of GAL4, which in turn recognizes UAS (CGGAGTACTGTCCTCCG) sites on the reporter plasmid DNA. Using this technique, CISAL-*BoxB* can be tethered to the 5×UAS sites on a reporter plasmid with the help of the λN–GAL4 fusion protein. The 5×*BoxB* DNA fragment with 5-ATATA-3 linking each other was synthesized from Generay Biotech (Shanghai, China). CISAL-*BoxB* was obtained by substituting the BRCA1 binding site in CISAL lncRNA with 5×*BoxB* using overlap PCR and cloned into a pcDNA3.1. The CISAL binding site in BRCA1 promoter cloned into pGL4.20 luciferase reporter vector was substituted with 5×UAS fragment to get pGL4-BRCA1-UAS using Q5 Site-Directed Mutagenesis Kit (NEB, Catalog # E0552). The GABPA recognizing site in pGL4-BRCA1-UAS was mutated to get pGL4-BRCA1-UAS-GABPA-mut.

Luciferase assay

A luciferase assay was carried out as previously described with modifications(Fan et al., 2015a). Briefly, pGL4-BRCA1-wt was obtained by cloning a 2200 bp DNA fragment (-2000 to +200 with respect to the BRCA1 transcriptional starting site) into the pGL4.20 vector upstream of the luciferase reporter gene. The mutant pGL4-BRCA1-mut plasmid was obtained by mutating the predicted CISAL binding motif. pGL4-BRCA1-GABPA-mut was generated by mutating the GABPA recognizing motif. MFF expression cassette containing miR-593-5p targeting site (wild type or mutated) was cloned into the pGL3-control plasmid downstream of the luciferase reporter gene, as previously described(Fan et al., 2015b). These pGL4.20 derivated reporter vectors were transfected into CAL-27 cells and the stable cell lines were obtained through puromycin selection for two weeks. CAL-27 cells stably expressing pGL4-BRCA1-wt or pGL4-BRCA1-mut were transfected with CISAL overexpressing vector or siRNAs using Lipofectamine 3000 (Invitrogen, Carlsbad, CA, USA). The pRL-TK plasmid delivering Renilla Luciferase was co-transfected as a control. The luciferase activities were measured using a Dual Luciferase Reporter Assay Kit (Promega, Madison, WI, USA), and the target effect was presented as the luciferase activity of the reporter vector with the target sequence relative to that without the target sequence.

Mitochondrial staining and analysis of mitochondrial fission

Mitochondrial staining was performed as described previously by us and other researchers with some modifications(Fan et al., 2015a; Fan et al., 2015b; Wang et al., 2011a). Briefly, cells were plated onto coverslips and treated as previously described. Then, the cells were stained for 30 min with 0.1 μM MitoTracker Red CMXRos (Molecular Probes). The mitochondria were visualized using a laser scanning confocal TCS SP5 microscope (Leica, Solms, Germany), and the mitochondrial morphology was assessed and quantified as described previously (Tanaka and Youle, 2008).

LncRNA expression profiles

CAL-27 and SCC-9 cells were treated with cisplatin (Sigma, USA) at its IC50(Fan et al., 2015a; Fan et al., 2015b) for 24 h for lncRNA microarray assays. Sample labeling and array hybridization were performed by Arraystar Human lncRNA Microarray V3.0 according to the Arraystar microarray-based gene expression analysis protocol.

mRNA profiles

CAL-27 and SCC-9 cells stably transduced with shRNA targeting CISAL were treated with cisplatin at the IC50 (Sigma, USA) for 24 h for mRNA profiling. The microarray data sets were normalized in GeneSpring GX using the Agilent FE one-color scenario.

Rapid amplification of cDNA ends (RACE)

RACE experiments were performed using the Smart RACE CDNA Amplification Kit (Clontech, Mountain View, CA) according to the manufacturer's instructions. Briefly, at least two sets of primers were designed and synthesized for nested PCR. The RACE PCR products were confirmed and separated by electrophoresis using a 1.5% agarose gel. Then, the amplified bands were sequenced. The CISAL sequence from the RACE analyses is listed in Supplementary Table 2. The gene-specific primers used for the nested PCR step of the RACE analysis of CISAL are listed in Supplementary Table 4.

RNA isolation and quantitative real-time PCR (qRT-PCR)

Total RNA was extracted using TRIzol reagent (Invitrogen, Waltham, MA, USA), and both nuclear and cytoplasmic RNA was isolated and purified with an RNA Purification Kit (Norgen, 21000-NB). cDNA was synthesized using M-MLV reverse transcriptase (Invitrogen, Waltham, MA, USA) according to the manufacturer's instructions (Invitrogen, Waltham, MA, USA). Then, qRT-PCR was performed using SYBR Green Real-time PCR Master Mix (ReverTra Ace, Toyobo) and a LightCycler 480 (Roche, Basel, Switzerland) according to the manufacturer's instructions. The PCR primers are listed in Supplementary Table 4. Ct values were calculated using the LightCycler 480 (Roche, Basel, Switzerland). The primers for hsa-miR-593-5p and U6 were purchased from RiboBio (Guangzhou, China).

Transfections

BRCA1 shRNAs (TRCN0000244987 and TRCN0000244986) were purchased from Sigma-Aldrich (St. Louis, USA), and scramble shRNA (#1864) was obtained from Addgene (MA, USA). GABPA Stealth Interference RNAs were purchased from Thermo Fisher Scientific. Sequences for these siRNAs are listed in Supplementary Table 5. Cells were plated in 6-well plates and transfected using Lipofectamine 3000 (Invitrogen, Waltham, MA, USA) according to the manufacturer's instructions.

Plasmid construction and establishment of stable cell lines

GABPA was amplified from cDNA using primers (Supplementary Table 4) and cloned into a pcDNA3.1 plasmid. Human full length BRCA1 was amplified from SFB-BRCA1 plasmid (#99394) purchased from Addgene (MA, USA) and cloned into

a pcDNA3.1 plasmid. DNA fragments (BRCA1-70 and BRCA1-120), including the complementary sequence to the predicted CISAL binding sites at (1134, 1157), were amplified from the plasmid delivering the BRCA1 promoter and then labeled with biotin using the Biotin 3' End DNA Labeling Kit (Thermo Fisher Scientific, #89818) according to the manufacturer's instructions. The primers used to amplify these DNA fragments are listed in Supplementary Table 4. The full length CISAL was amplified from cDNA using primers and cloned into a pcDNA3.1. The site of CISAL binding to BRCA1 promoter was mutated to get mut-CISAL. A series 5' deletion mutants (1-1300, 1-1100, 1-900, 1-700, 1-500, 1-300) and antisense of CISAL were amplified from wt-CISAL and cloned into a pcDNA3.1. All site-specific mutants were obtained using the QuikChange II Site-Directed Mutageneration Kit (Agilent, Catalog #200523). shRNAs targeting CISAL were constructed using the pSIREN-RetroQ-DsRed-Express retrovirus vector from Takara Biotechnology (Dalian, China). The CISAL siRNA target sequences and control sequences are listed in Supplementary Table 5. To generate stable TSCC cell lines with CISAL knockdown or overexpression, pSIREN-RetroQ-DsRed-Express or pBABE-puro based construct was co-transfected with helper plasmids into HEK293T cells to produce recombinant viruses. Infection and screening of TSCC stable cells were performed as we have described previously(Fan et al., 2015a; Fan et al., 2015b).

Bioinformatic analysis

Three different methods including open reading frame finder from NCBI (Kozak strength)(Nishikawa et al., 2000), txCdsPredict from UCSC and phyloCSF(Lin et al., 2011) were performed to calculate the coding potential of CISAL. Defining txCdsPredict: 800(Prensner et al., 2011) as thresholds, the scores of open reading frames from CISAL were well below the thresholds as indicating as txCdsPredict scores=285. Non coding frame was detected by phyloCSF(Cabili et al., 2011) as scores≤0.

The potential binding sites between CISAL and BRCA1 or GAPDH were predicted by IntaRNA (http://rna.informatik.uni-freiburg.de/IntaRNA/Input.jsp). The base-pairing energy for an RNA/RNA duplex model of seed-based regions along the CISAL transcript and 2,000 bp upstream and 200 bp downstream of the TSS of BRCA1 or GAPDH was calculated(Grote et al., 2013). The duplex energy was computed for each such region and displayed in a heatmap. The probabilities were then averaged for a sliding window of seed-based regions to give the average RNA

accessibility of the binding region. The average probability of single-stranded RNA was computed using a Sfold server (http://sfold.wadsworth.org)(Ding et al., 2004).

Entrez cancer gene list was established as previously described(Domazet-Loso and Tautz, 2010). Cancer associated TFs were defined by overlapping Entrez cancer genes and TFs with ChIP-seqs in Encyclopedia of DNA Elements (ENCODE). We chose TFs ChIP-seqs from GM12878, K562, HepG2 and Hela-S3 cell lines and downloaded them from ENCODE. We then processed them by ENCODE processing pipeline and the TFs binding sites at BRCA1 promoter (NG_056086.1) were analyzed. To predict the potential GABPA binding site at BRCA promoter region, we used motif-counter (https://bio.tools/motifcounter) to scan BRCA promoter region both strand with GABPA motif which was downloaded from JASPAR database.

Northern blotting

Northern blots were performed as previously reported with some modifications(Kim et al., 2016; Nakamura et al., 2001). Briefly, 10 μg of RNA was fractionated on a 1% agarose gel at 50 V for 1.5 h. The RNA was then transferred to a nitrocellulose membrane (Millipore, HATE00010) overnight for at least 16 h. After being washed with 2× SSC, the nitrocellulose membrane was UV crosslinked at 1,000 μF and prehybridized for 30 min at 52°C in UltraHyb buffer (Roche, 11796895001). Then, DIG-labeled probes were added, and the blots were hybridized in 10 mL of UltraHyb buffer. After being washed for 2×5 min at the hybridization temperature in low-stringency buffer and for 2×15 min at the hybridization temperature in high-stringency buffer, the blots were incubated for 30 min in blocking solution and then for 30 min in anti-digoxigenin-AP solution and analyzed on a phosphorimager (Molecular Dynamics) with a CSPD detection buffer.

RNA fluorescence in situ hybridization (FISH)

Cells were washed with PBS and fixed with 4% formaldehyde in PBS (pH 7.4) for 20 min at room temperature. Then, the cells were permeabilized in PBS containing 0.1% Triton X-100 at room temperature for 15 min, washed with PBS for 3×5 min, digested with 0.05% trypsin, and subjected to dehydration in an ethanol series (75%, 85% and 100%) prior to hybridization. Hybridization was performed using a DIG-labeled CISAL probe in hybridization solution (probe dilution 1:1000) (Exiqon, Denmark) for 16 h at 52°C in a humidified chamber. Next, the cells were washed for 30 min in 2× SSC at 52°C and then for 30 min in 25% deionized formamide/2× SSC at 52°C. Cells on coverslips were counterstained with DAPI and imaged using a TCS SP5 confocal

microscope (Lecia, Solms, Germany).

Western blotting

Immunoblotting was performed as previously described (Fan et al., 2015a). Briefly, cells were lysed for 0.5 h at 4°C in RIPA Buffer (R0278, Sigma) containing a protease inhibitor cocktail. Protein extracts were resolved through 8% SDS-polyacrylamide gel electrophoresis; transferred to polyvinylidene difluoride membranes (BioRad, Berkeley, CA, USA); probed with antibodies against human MFF (ab81127, Abcam), BRCA1 (ab191042, Abcam), GABPA (Millipore, ABE1047), PCNA (ab92552, Abcam) and β-actin (Proteintech, Chicago, IL, USA) and then with a peroxidase-conjugated secondary antibody (Proteintech); and finally visualized via chemiluminescence (GE, Fairfield, CT, USA).

Immunofluorescence staining

Cells on coverslips were stained for mitochondria and cytochrome c(CYT c). After mitochondrial staining, the cells were incubated with primary antibodies against CYT c (Santa Cruz, sc-13560) and then incubated with FITC-conjugated secondary antibodies (Invitrogen). The coverslips were counterstained with 46-diamidino-2-phenyl indole and imaged under a confocal microscope TCS SP5 (Lecia, Solms, Germany).

Apoptosis assay

For apoptosis assays, CAL-27 and SCC-9 cells were treated with cisplatin under 8×10^{-6} and 1.8×10^{-5} M (Sigma, USA) for 24 h(Fan et al., 2015a). Apoptosis was detected using TUNEL, flow cytometry and caspase-3/7 activity assays. TUNEL assays were performed using a kit from Roche (Cat. No. 11684795910) according to the user's instructions. Sections were examined with an ImagerZ1 microscope (Zeiss, Jena, Germany). An investigator blinded to the treatment quantified 20 random fields from the samples. Flow cytometry was performed using Annexin V and propidium iodide double staining (Sigma-Aldrich). Caspase-3/7 activity was determined using an Apo-ONE® Homogeneous Caspase-3/7 Assay Kit from Promega according to the manufacturer's protocol.

In situ hybridization (ISH)

In situ hybridization was performed as previously described(Fan et al., 2015a) according to the manufacturer's protocol (Exiqon, Vedbaek, Denmark). Briefly, after demasking, CISAL was hybridized to 5'DIG-labeled CISAL probes. Then, DIG was recognized via a specific anti-DIG antibody directly conjugated to alkaline

phosphatase. The nuclei were counterstained with Kernechtrot Solution (N3020, Sigma). In all, 5×200 tumor cells were counted randomly in each section. Sections with more than 300 RNA-positive cells were considered to have high expression.

Immunohistochemistry (IHC)

Immunohistochemistry was performed as described previously(Fan et al., 2015a). Briefly, slice of paraffin-embedded tissues were deparaffinized, rehydrated and subjected to antigen retrieval. Then, the tissues were incubated with anti-BRCA1 (ab16780, Abcam) antibody at 4 °C overnight and then successively incubated with secondary antibody and streptavidin–horseradish peroxidase complex. Diaminobenzidine (Dako, Carpinteria, CA, USA) was used as a chromogen, and the nuclei were counterstained with hematoxylin. In total, 5×200 tumor cells were counted in each section. Sections with more than 300 BRCA1-positive cells were considered to have high BRCA1 expression.

Tumor xenografts

Male BALB/c nude mice aged 4 to 6 weeks were prepared for tumor implantation. CAL-27 cells (5×10^6/mouse) stably expressing CISAL or shRNA targeting CISAL were resuspended in 150 µL of PBS and injected subcutaneously into the flanks of the nude mice. One week after implantation, when the tumor became palpable at ~2 mm in diameter, either cisplatin or saline were intraperitoneally injected at 5 mg/kg body weight every three days from days 8 to 32. Tumor volume was calculated beginning at the first day of cisplatin injection using the formula TV (mm^3)=length×width^2×0.5. At day 35, the primary tumors were carefully removed for analysis as indicated.

Patient and tissue samples

Fresh tumor tissues from 29 TSCC patients for identification of lncRNA profiles were obtained before and after neoadjuvant chemotherapy while specimens from 113 locally advanced TSCC patients were obtained before neoadjuvant chemotherapy between Jan 1, 2004, and Dec 31, 2010. Patients with locally advanced resectable TSCC (stage III or IVA) underwent one or two cycles of neoadjuvant chemotherapy(Zhong et al., 2013) (75 mg/m^2 cisplatin on day 1, 75 mg/m^2 docetaxel on day 1, and 750 mg/m^2 fluorouracil on days 1 to 5), and the tumor response to neoadjuvant chemotherapy was assessed by CT/MRI studies prior to radical resection. According to the Response Evaluation Criteria in Solid Tumors of the World Health Organization, TSCC patients with progressive or stable disease were characterized as having nonsensitive TSCC, whereas those who showed a partial or complete response

were determined to have cisplatin-sensitive TSCC. Surgery was performed at least 2 weeks after the completion of neoadjuvant chemotherapy. Histological diagnoses and scoring of all the cases were performed by two independent pathologists. The survival time was calculated from the date of surgery to the date of death or last follow-up. The date of death was obtained from patient records or through follow-up telephone calls.

Study approval

Ethical consent was given by the Sun Yat-sen University Committee for Ethical Review of Research Involving Human Subjects. Human TSCC were obtained with written and informed consent under 2014114 from Sun Yat-sen Memorial Hospital. The animal experiments were in accordance with the institutional authorities' guidelines and formally approved by the Animal Ethics Committee of Sun Yat-sen University.

Statistics

All data are presented as mean ± SEM. Comparisons between 2 groups were done by 2-tailed Student's t tests using the SPSS 18.0 package (SPSS, Chicago, IL, USA). For multiple comparisons between groups, 1-way ANOVA followed by Dunnett's multiple comparisons tests was performed. A chi-square test were used to analyze the relationship between CISAL and BRCA1 expression and the clinicopathological characteristics using the SPSS 18.0 package. To measure associations between pairs of variables, Spearman order correlations were performed. Kaplan-Meier survival curves were plotted, and a log-rank test was performed. At least three independent experiments were performed for all cell culture experiments. Throughout the study, P values below 0.05 were considered significant.

Data availability

Data have been deposited in the Gene Expression Omnibus (GEO) DataSets (https://www.ncbi.nlm.nih.gov/gds) under the following accession numbers: GSE114929 and GSE115116.

Supplemental Reference

Cabili, M.N., Trapnell, C., Goff, L., Koziol, M., Tazon-Vega, B., Regev, A., and Rinn, J.L. (2011). Integrative annotation of human large intergenic noncoding RNAs reveals global properties and specific subclasses. Genes & development *25*, 1915-1927.

Chu, C., Quinn, J., and Chang, H.Y. (2012). Chromatin isolation by RNA purification (ChIRP). Journal of visualized experiments : JoVE.

Ding, Y., Chan, C.Y., and Lawrence, C.E. (2004). Sfold web server for statistical folding and rational design of nucleic acids. Nucleic acids research *32*, W135-141.

Domazet-Loso, T., and Tautz, D. (2010). Phylostratigraphic tracking of cancer genes suggests a link to the emergence of multicellularity in metazoa. BMC biology *8*, 66.

Fan, S., Chen, W.X., Lv, X.B., Tang, Q.L., Sun, L.J., Liu, B.D., Zhong, J.L., Lin, Z.Y., Wang, Y.Y., Li, Q.X.*, et al.* (2015a). miR-483-5p determines mitochondrial fission and cisplatin sensitivity in tongue squamous cell carcinoma by targeting FIS1. Cancer letters *362*, 183-191.

Fan, S., Liu, B., Sun, L., Lv, X.B., Lin, Z., Chen, W., Chen, W., Tang, Q., Wang, Y., Su, Y.*, et al.* (2015b). Mitochondrial fission determines cisplatin sensitivity in tongue squamous cell carcinoma through the BRCA1-miR-593-5p-MFF axis. Oncotarget *6*, 14885-14904.

Grote, P., Wittler, L., Hendrix, D., Koch, F., Wahrisch, S., Beisaw, A., Macura, K., Blass, G., Kellis, M., Werber, M.*, et al.* (2013). The tissue-specific lncRNA Fendrr is an essential regulator of heart and body wall development in the mouse. Developmental cell *24*, 206-214.

Kim, Y.K., Kim, B., and Kim, V.N. (2016). Re-evaluation of the roles of DROSHA, Export in 5, and DICER in microRNA biogenesis. Proceedings of the National Academy of Sciences of the United States of America *113*, E1881-1889.

Lin, M.F., Jungreis, I., and Kellis, M. (2011). PhyloCSF: a comparative genomics method to distinguish protein coding and non-coding regions. Bioinformatics *27*, i275-282.

Nakamura, K., Robertson, M., Liu, G., Dickie, P., Nakamura, K., Guo, J.Q., Duff, H.J., Opas, M., Kavanagh, K., and Michalak, M. (2001). Complete heart block and sudden death in mice overexpressing calreticulin. The Journal of clinical investigation *107*, 1245-1253.

Nishikawa, T., Ota, T., and Isogai, T. (2000). Prediction whether a human cDNA sequence contains initiation codon by combining statistical information and similarity

with protein sequences. Bioinformatics *16*, 960-967.

Prensner, J.R., Iyer, M.K., Balbin, O.A., Dhanasekaran, S.M., Cao, Q., Brenner, J.C., Laxman, B., Asangani, I.A., Grasso, C.S., Kominsky, H.D.*, et al.* (2011). Transcriptome sequencing across a prostate cancer cohort identifies PCAT-1, an unannotated lincRNA implicated in disease progression. Nature biotechnology *29*, 742-749.

Schmitz, K.M., Mayer, C., Postepska, A., and Grummt, I. (2010). Interaction of noncoding RNA with the rDNA promoter mediates recruitment of DNMT3b and silencing of rRNA genes. Genes & development *24*, 2264-2269.

Tanaka, A., and Youle, R.J. (2008). A chemical inhibitor of DRP1 uncouples mitochondrial fission and apoptosis. Molecular cell *29*, 409-410.

Wang, J.X., Jiao, J.Q., Li, Q., Long, B., Wang, K., Liu, J.P., Li, Y.R., and Li, P.F. (2011a). miR-499 regulates mitochondrial dynamics by targeting calcineurin and dynamin-related protein-1. Nature medicine *17*, 71-78.

Wang, K.C., Yang, Y.W., Liu, B., Sanyal, A., Corces-Zimmerman, R., Chen, Y., Lajoie, B.R., Protacio, A., Flynn, R.A., Gupta, R.A.*, et al.* (2011b). A long noncoding RNA maintains active chromatin to coordinate homeotic gene expression. Nature *472*, 120-124.

Wei, M., Grushko, T.A., Dignam, J., Hagos, F., Nanda, R., Sveen, L., Xu, J., Fackenthal, J., Tretiakova, M., Das, S.*, et al.* (2005). BRCA1 promoter methylation in sporadic breast cancer is associated with reduced BRCA1 copy number and chromosome 17 aneusomy. Cancer research *65*, 10692-10699.

Zhong, L.P., Zhang, C.P., Ren, G.X., Guo, W., William, W.N., Jr., Sun, J., Zhu, H.G., Tu, W.Y., Li, J., Cai, Y.L.*, et al.* (2013). Randomized phase III trial of induction chemotherapy with docetaxel, cisplatin, and fluorouracil followed by surgery versus up-front surgery in locally advanced resectable oral squamous cell carcinoma. Journal of clinical oncology : official journal of the American Society of Clinical Oncology *31*, 744-751.
